# Pan-immune system, mobilome and resistome in Streptococcus suis

**DOI:** 10.1099/mgen.0.001521

**Published:** 2025-09-29

**Authors:** Manon Dechêne-Tempier, Stéphanie Bougeard, Valentin Loux, Hélène Chiapello, Virginie Libante, Corinne Marois-Créhan, Nathalie Leblond-Bourget, Sophie Payot

**Affiliations:** 1 Anses Laboratoire de Ploufragan-Plouzané-Niort, Unité Mycoplasmologie, Bactériologie et Antibiorésistance, BP53, 22440 Ploufragan, France; 2 Université de Lorraine, INRAE, DynAMic, F-54000 Nancy, France; 3 Anses Laboratoire de Ploufragan-Plouzané-Niort, Unité Épidémiologie, santé et bien-être, BP53, 22440 Ploufragan, France; 4 MaIAGE, Université Paris-Saclay, INRAE, Jouy-en-Josas, France; 5 BioinfOmics, MIGALE Bioinformatics Facility, Université Paris-Saclay, INRAE, Jouy-en-Josas, France

**Keywords:** antimicrobial resistance genes, competence, CRISPR, defence systems, integrative and conjugative elements, integrative and mobilizable elements, restriction–modification systems, *Streptococcus suis*, virulence

## Abstract

*Streptococcus suis* is a bacterial pathogen responsible for infections in pigs and in wild fauna that can also lead to severe infections in humans. Increasing antimicrobial resistance (AMR) has been described for this zoonotic pathogen worldwide. Since most of these AMR genes are carried by mobile genetic elements (MGEs), they can largely disseminate by horizontal gene transfer. Taking advantage of the large set of genomes available for this species, an exhaustive search of integrative and conjugative elements (ICEs) and integrative and mobilizable elements (IMEs) was undertaken in a representative set of 400 selected high-quality genomes of *S. suis*. We examined how these elements vary across phylogenetic clades and ecotypes and their association with AMR genes and defence systems (DSs), including restriction–modification (RM), CRISPR and also less studied DSs. This investigation identified 569 ICEs, belonging to the 7 families previously described in streptococci, inserted in 12 distinct specific integration sites. Additionally, 1,035 IMEs characterized by 11 distinct relaxase families and integrated in 10 specific chromosomal sites were detected in the 400 genomes of *S. suis*. New associations between ICE/IME and AMR genes were discovered. A huge diversity of putative DSs was observed including 2,035 RM systems, 124 CRISPR systems and systems belonging to 20 other categories, most of them described as efficient against phages and plasmids. Furthermore, most of the spacers associated with CRISPR systems target these MGEs rather than integrative elements. In addition, many integrative elements appear to carry an orphan methylase that could help them escape RM systems. Altogether, this points out that ICEs and IMEs are spared by DSs and play a major role in AMR dissemination in *S. suis*. In addition, most of the strains have the full set of genes required for competence, i.e. for the acquisition of extracellular DNA by natural transformation. This suggests a high risk of AMR dissemination in *S. suis*.

**Impact Statement**

As a major disease agent in pigs, the zoonotic pathogen *Streptococcus suis* raises economic and public health concerns. Increasing antimicrobial resistance (AMR) and carriage of the AMR determinants by mobile genetic elements have been reported earlier for this species. Using publicly available genomes, we built a representative set of 400 high-quality genomes of *S. suis* that could also be useful for other scientific studies. Analysis of this set provided a more comprehensive insight of the diversity and distribution of integrative and conjugative elements (ICEs) and integrative and mobilizable elements (IMEs) and their association with AMR genes in *S. suis*. We also conducted the first exhaustive analysis of defence systems (restriction–modification, CRISPR and also less studied defence systems) in this species. A high diversity of putative defence systems was detected, predominantly targeting phages rather than integrative elements. These latter seem to be armed to escape restriction–modification systems, possibly explaining their high prevalence in *S. suis*. New ICE/IME-AMR gene associations were discovered. In addition, most of the strains could acquire foreign DNA by natural transformation. AMR genes are widespread in carriage strains of *S. suis*. A surveillance of AMR in *S. suis* that includes carriage strains is thus needed.

## Data Summary

The authors confirm that all supporting data, code and protocols have been provided within the article or through supplementary data files.

Genome assemblies of strains 1112, 1111, 1108 and 1102 have been deposited at DDBJ/ENA/GenBank under the accession numbers JBBJJD000000000, JBBJJE000000000, JBBJJF000000000 and JBBJJG000000000, respectively (see Table S1, available in the online Supplementary Material).

## Introduction

*Streptococcus suis* can cause opportunistic systemic infections, such as meningitis and septicaemia in pigs, leading to high economic losses. This pathogen is also zoonotic and can be responsible for severe infections such as meningitis and sepsis in humans [1]. Many virulence or virulence-associated factors have been characterized in *S. suis,* but predicting its pathogenic potential based solely on the presence or absence of those factors remains challenging. Indeed, infections caused by *S. suis* likely depend on multiple factors, in particular host immunity and stress, and polymicrobial infections [2,6]. A total of 29 serotypes has been defined for this bacterial species on the basis of the antigenicity of capsular polysaccharides [7]. Serotype 2 is the most common cause of infections in pigs and humans, but other serotypes are of increasing importance, in particular serotype 9 in some European countries [2,8]. In addition, many isolates that circulate are nontypeable due to new variants of the *cps* locus [9,10]. *S. suis* can also be genetically differentiated using multilocus sequence typing (MLST), and ST1 strains are frequently associated with disease in both pigs and humans worldwide [2].

Thanks to the increase of available sequenced genomes in the last decade, methods based on pangenome analysis rather than on a set of genes are now frequently used to depict the overall genomic diversity of bacterial species [11,12]. Pangenomes are composed of a core genome, i.e. a set of genes that are shared by all genomes of a taxonomic unit and an accessory variable genome corresponding to a collection of genes found in some but not all genomes. The core genome is generally used to group strains and infer phylogenetic clades, while the accessory genome is crucial to understand the specific lifestyle, adaptation patterns and evolutionary trajectories of the different strains and clades. Several divergent clades have been described in *S. suis*, but phenotypic similarities and horizontal gene transfer (HGT) with the core population hinder their assignment to new sub-species or species [13,14]. The pangenome of *S. suis* is considered to be open, and HGT is likely a major driving force for genome diversification [15].

Chromosomal mobile genetic elements (MGEs), in particular elements transferring by conjugation, play a major role in HGT in *S. suis* [16,21]. Integrative and conjugative elements (ICEs) are chromosomal MGEs that can excise, autonomously transfer by conjugation and integrate in the chromosome of the recipient cell [22,23]. In addition to the genes involved or controlling their mobility, ICEs carry cargo genes which can provide advantageous properties to their bacterial host including stress response, catabolic properties, virulence and antibiotic resistance. Other MGEs called integrative and mobilizable elements (IMEs) encode their own excision and integration in the bacterial chromosome but hijack the conjugative machinery of conjugative elements to transfer by conjugation. These mobile elements have been less studied than ICEs because they are more difficult to identify in bacterial genomes, due to their size, the small number of specific signature proteins and the fact that they are frequently part of composite elements, i.e. in tandem with other MGEs after accretion or as nested elements after integration inside other MGEs [24,25]. IMEs also largely participate in the acquisition and dissemination of antimicrobial resistance (AMR) genes in *S. suis* [16,18]. *S. suis* can also capture extracellular DNA and integrate this DNA in its chromosome by natural transformation. This mechanism of gene capture involves several sequential steps before incorporation of the foreign DNA in the bacterial chromosome by homologous recombination [26].

Interactions between MGEs and their bacterial host can range from mutualistic to parasitic. Hence, bacteria need defence systems (DSs) in order to fight against detrimental exogenous MGEs. Knowledge on the bacterial arsenal of DSs has greatly increased in the past years [27,30]. Bacteria frequently encode multiple DSs. DSs can be classified according to their mode of action: (i) degradation of the genetic material of the invading agent, (ii) inhibition of DNA or RNA synthesis and (iii) induction of growth arrest or cell suicide, for a protection at the population rather than individual level [29]. The first mechanism is the most widespread with restriction–modification (RM) systems and clustered regularly interspaced short palindromic repeats (CRISPRs)-CRISPR-associated (Cas) proteins present in 95% and 42% of bacterial genomes, respectively [29]. Four types of RM systems (I–IV) have been defined on the basis of the subunit composition and biochemical characteristics of the systems [29,30]. RM systems need two components: a methyltransferase that methylates adenines or cytosines within a specific sequence context and a restriction endonuclease that recognizes and cleaves the same motif lacking methylation. The exception is type IV RM systems that cleave methylated instead of unmethylated DNA. Type I RM systems have separated subunits, called S subunits, to impart specificity to both the restriction and modification activities of the complex. CRISPR-Cas systems include one or more CRISPR arrays, i.e. short sequences called spacers complementary to specific sequences of MGEs and separated by short repeats and functionally associated *cas* genes. CRISPR-Cas immunity involves three steps: processing of fragments of MGEs and incorporation as spacers in the array, transcription and maturation of spacers and detection-destruction of invading RNA or DNA complementary to the spacer by an interference complex formed by the mature CRISPR RNA and Cas proteins [29,30]. Other less frequent DSs leading to the degradation of the invading DNA include the Gabija, Nhi and Kiwa systems [29,30]. The second group of DSs interferes with DNA replication or transcription through the depletion of DNA or RNA nt, e.g. Hachiman systems, transmembrane NTPase (Tmn), restriction by an adenosine deaminase acting on RNA and defence-associated reverse transcriptase (DRT) [29,30]. The third group of DSs acts at the level of the population, the most common ones being abortive infection (Abi) systems that include unrelated systems having in common their mechanism of action whereby cells die upon viral or plasmid invasion thus preventing the spread. Many systems involve intracellular signalling to trigger an Abi response [29,30]. This is the case for the cyclic-oligonucleotide-based antiphage signalling systems (CBASSs) and the Thoeris system. Some systems are associated with a reverse transcriptase (RT), like retrons that are tripartite systems formed by a RT, a non-coding RNA and accessory effector proteins. Other systems like antiviral ATPases/NTPases of the STAND superfamily (AVAST) have a conserved tripartite organization, composed of a C-terminal sensor domain that recognizes specific viral targets, a core NTPase domain and an N-terminal effector domain. Three other systems also protect bacteria from viral and plasmid infection by triggering an Abi response [29,30]: the Lamassu family that involves proteins of the structural maintenance of chromosomes (SMC) family, the PARIS family that detects phage anti-restriction protein and the PrrC protein that monitors the normal activity of the type I restriction enzyme EcoprrI. It is worth mentioning that DSs are frequently clustered in genomic regions called ‘defence islands’ [31] that may themselves be located on MGEs, thus enabling their horizontal transfer and contribution to competition between MGEs [32]. Knowledge on DSs is scarce in *S. suis*. Only one study, conducted on 58 genomes, reported on RM, CRISPR and Abi systems [33]. In this work, an exhaustive analysis of DSs was undertaken in a large set of high-quality genomes covering the diversity of the *S. suis* species. Mobilome and resistome were analysed in order to study if there is any correlation with the DS arsenal of the strains.

## Methods

### DNA extraction and whole-genome sequencing

DNA extraction, whole-genome sequencing and assembly as well as quality checking of the reads and the assembly were done as described previously [18].

### Genomic analyses

RefSeq genomes [34] available for the *S. suis* species were downloaded from the NCBI database using the NCBI Datasets command-line tools datasets and dataformat [35]. At the time this work was initiated (July 2022), 2,429 RefSeq genomes were retrieved using genome taxon parameter 1307. Additional genomes of *S. suis* sequenced by Anses Ploufragan (*n*=159), not publicly available in the NCBI database at the beginning of the work, were added to this initial set to reach a total of 2,588 genomes.

To keep only high-quality representative genomes and discard the too closely related ones, we used the dRep bioinformatic tool (3.2.2 version) [36], using the dereplicate option with an average nt identity (ANI) of 97.5% for primary clustering and 99% for secondary clustering and two filters based on checkM score criteria [37]: a maximum of 5% of contamination and a minimum of 99% for completeness. An additional quality criterium proposed by Bortolaia *et al*. [38] was applied (N50 >30 kb). Genomic relatedness of the final set of strains was analysed using dRep 3.2.2 through the determination of the ANI without primary clustering by using the dRep compare option. Twenty genomes sequenced by Anses Ploufragan were selected in the winner set of high-quality genomes. Sixteen, corresponding to French isolates, were published in 2024 [18]. The other four genomes correspond to re-sequenced genomes of reference strains (see Table S1) and were publicly released under the NCBI accession numbers JBBJJD000000000, JBBJJE000000000, JBBJJF000000000 and JBBJJG000000000 at the end of this work.

Metadata (host, isolation country, collection year and isolation site of the strain) were collected for the representative set of genomes on the basis of submitted GenBank files or by manually extracting data in published papers when information was missing in the files. When information was not found, ‘unknown’ was indicated. Strains were classified in five isolation groups: upper respiratory tract (when the following terms were indicated for the sample description: ‘tonsil’, ‘nasal cavity’, ‘clinically healthy’ or ‘avirulent’), lower respiratory tract (when the following terms were indicated for the sample description: ‘respiratory tract’ or ‘lung’), systemic (when the following terms were indicated for the sample description: ‘systemic’, ‘gastrointestinal tract’, ‘brain’, ‘blood’, ‘brain or blood’, ‘nervous system’, ‘lymphatic node’ or ‘diseased’), environment (when the following term was indicated for the sample description: ‘farm’) and unknown.

For incomplete genomes (available in RefSeq as a list of contigs), scaffolding was done using the Multi-Draft based Scaffolder MeDuSa [39] with a set of 21 complete reference genomes of *S. suis* to generate pseudochromosomes as described previously [18]. Remaining contigs were concatenated at the end of the pseudochromosome using Geneious Prime software (version 2023.2.1, Biomatters). This scaffolding is highly recommended for the analysis of ICEs and IMEs using ICEscreen (see below). Complete genomes and pseudochromosomes generated in the previous step were then annotated with Prokka [40] using the reference strain BM407 (RefSeq accession number: GCF_000026745.1).

MLST was done using the pipeline developed by Athey *et al*. [41]. MLST groups were attributed using the scheme developed by King *et al*. [42] and the webtool pubMLST (https://pubmlst.org/) [43].

The serotype was determined by local custom blast interrogation of a home-built database with the megablast program (*E*-value cutoff 1×10^−20^) using the Geneious Prime software (version 2023.2.1, Biomatters). Distinction between serotypes 1-14 and 2-1/2 was done by analysing the *cpsK* gene. The sequences of the *cps* loci used for this analysis are given in File S1.

Annotated genomes (complete genomes and pseudochromosomes) were used as inputs for analysis by PPanGGOLiN (version 2.0.2) [12] to construct the pangenome and extract and align persistent genes (using mafft as alignment tool and DNA as source with the –source dna option). The persistent genome represents genes found in the majority of genomes, usually more than 90%. It is conceptually similar to the core genome, but its definition is more tolerant to gene loss events, such as those resulting from assembly gaps [12]. Similar results were obtained for incomplete genomes when using pseudochromosomes or multifasta files as inputs for PPanGGOLiN. Results were concatenated in a single multifasta file using the –phylo option of PPanGGOLiN. This nt alignment was then used to generate a maximum likelihood phylogenetic tree of the strains using FastTree 2.1.10 [44] with 1,000 bootstrap replications. One of the two divergent clades was used as an outgroup to obtain an outgroup-rooted tree. The tree was visualized and annotated using the R library ggtree [45].

A total of 70 genetic markers associated with virulence were searched by local custom blast with the blastx program (*E*-value cutoff 1×10^−20^) using the Geneious Prime software (version 2023.2.1, Biomatters) as described previously [18].

The search for AMR genes was done by local custom blast with the blastn program (*E*-value cutoff 1×10^−20^) using the Geneious Prime software (version 2023.2.1, Biomatters), against the imported ResFinder 4.1 [46] and the CARD 3.0.3 database [47]. Other AMR variants previously described in *S. suis* were searched as described previously [18]. A hit was retained only if it shows more than 70% of nucleic acid identity with the query, with at least 80% of coverage of the query. The genetic environment of the AMR genes was analysed using the Geneious Prime software (version 2023.2.1, Biomatters). To determine if AMR genes are localized on an ICE or an IME, we first extracted the AMR genes located up to 60 kb upstream or downstream of a relaxase gene (using the positions indicated in the output ‘*_detected_ME.tsv’ file of ICEscreen) using a custom R script and then manually checked if these AMR genes are located on MGEs by using the output annotated files generated by ICEscreen [48].

Coding DNA sequences (CDS) annotations, corresponding to the translated aa sequence with indication of the locus tag, its position in the genome and its description, were extracted from the genomes using Geneious Prime software (version 2023.2.1, Biomatters). Multifasta files were then used for searching RM systems by local custom blast interrogation of the REBASE Gold database (https://rebase.neb.com/cgi-bin/rebgoldlist, last interrogation 02/2021) using the blastp program (*E*-value cutoff 1×10^−30^). A hit was retained only if it shows more than 80% of coverage of the query. Information about the category of RM was obtained from the REBASE enzyme database (https://rebase.neb.com/rebase/rebase.enz.html). Manual curation was done in order to eliminate multiple occurrences of the same RM system (as a result of multiple subunits of the same RM system). Only full RM systems were counted, which means (i) for type I RM, at least one specificity subunit, one restriction subunit and one methylase subunit, (ii) for types II and III RM systems, one restriction subunit and one methylase subunit and (iii) two subunits for type IV. A methyltransferase was considered as ‘orphan’ if no cognate restriction endonuclease was found at a distance of less than ten genes away. RM systems were also analysed by using PADLOC (version 1.1.0) [49].

CRISPR systems were searched by three methods: (i) by local custom blast interrogation of a home-built database consisting of sequences of Cas proteins previously reported in *S. suis* [33] (see File S2) with the blastp program (*E*-value cutoff 1×10^−20^) using the Geneious Prime software (version 2023.2.1, Biomatters), (ii) using CRISPR-Cas finder, with default parameters except the minimal repeat length that was decreased to 19 [50] and (iii) using PADLOC (version 1.1.0) [49]. Spacers included in the arrays of CRISPR-Cas systems were analysed by the blastn program against the NCBI database (last interrogation: July 2024) to see if they match sequences of phages, plasmids, ICEs or other MGEs.

Annotated genomes (complete genomes and pseudochromosomes) were used as inputs for the ICEscreen tool (version 0.4, https://icescreen.migale.inrae.fr/) [48] in order to identify chromosomal integrative elements. Elements encoding the four signature proteins of ICEs [integrase, coupling protein (CP), relaxase and VirB4] were counted as putative ICEs and those with at least one pseudogene as defective ICEs. Likewise, elements were considered as putative IMEs if they encode at least an integrase and a relaxase and as defective IMEs if at least one of these genes is a pseudogene. When elements were present on multiple contigs separated by a gap, they were indicated as ‘partial’.

As described previously [18], competence was analysed by searching 38 genes in the genomes by local custom blast interrogation of a home-built database using the blastx program (*E*-value cutoff 1×10^−20^), and a pherotype was attributed to the strains on the basis of the ComS sequence.

### Statistical analyses and data visualization

Due to the large number of variables, principal component analysis (PCA) (for quantitative variables) or multiple correspondence analysis (MCA) (for qualitative variables) followed by hierarchical clustering on principal components (HCPC) analysis was first used to identify the most discriminatory genetic markers and decrease the number of variables. Variables with less than 5 hits were not included in the analysis. To decrease the number of modalities per qualitative variable for host and serotypes, the less frequent ones were grouped. For quantitative variables (number of ICEs, number of IMEs, number of ICEs+IMEs, number of AMR genes, number of virulence markers, genome length, number of orphan methylases, number of RM systems, number of DSs, number of Cas systems and number of spacers), we calculated the median (corresponding to the middle 50% of the data dispersion) and 25^th^ (Q1) and 75^th^ (Q3) percentiles (corresponding to the thresholds of 25% and 75% of the data dispersion) and defined three groups: <Q1, in interquartile range (IQR i.e. [Q1-Q3]) and >Q3.

Chi-square tests (with Fisher correction for limited expected counts) were used to study the associations between the various genomic traits of the strains. Only results with a *P*-value equal to or lower than 0.05 (i.e. 95% of level of confidence) were considered significant.

PCA, MCA, HCPC analysis and chi-square tests (with Fisher correction for limited expected counts) were done using R (FactoMineR version 2.8 package with the PCA, MCA and HCPC functions and chisq.test (fisher.test), respectively). Figures were made using the ComplexHeatmap 2.16.0 [51], ggplot2 [52], UpsetR 1.4.0 [53] packages of R, EasyFig 2.2.5 (https://www.beatsonlab.com/softwares/easyfig/) or Geneious Prime software (version 2023.2.1, Biomatters).

## Results

A set of 400 high-quality genomes that includes 29 complete genomes and 371 draft genomes of *S. suis* and covers the genomic diversity of the species was obtained by using the dRep tool. The corresponding strains were mostly isolated from pigs (*n*=387), and six strains were isolated from environmental sources, three from humans (strains 39565, 861160 and GX69), two from wild boars (strains 1124 and 1450) and two from cattle (strains 86-5192 and 10-36905) (Table S1). Strains arose from 3 continents and 11 countries, mostly from China (*n*=167) followed by UK (*n*=116), Canada (*n*=72), France (*n*=16), the Netherlands (*n*=8) and USA (*n*=7). Strains were also collected from other countries from Europe (four from Denmark, four from Switzerland and two from Germany) and Asia (two from Thailand and two from Japan) (classified as ‘other’ in Table S1 because of less than five strains). The set of 400 genomes includes 143 strains isolated from the upper respiratory tract, 62 strains responsible for systemic infections, 18 isolates from the lower respiratory tract and 6 from the environment. Information about the isolation site is missing for 171 strains (see Table S1). Twenty-three strains were isolated before 2001, 8 during years 2001–2005, 32 in the 2006–2010 period, 175 in the 2011–2015 period and 107 more recently (2016–2020). Information about the year of strain isolation is missing for 55 strains (Table S1). All the 30 known serotypes, i.e. the 29 serotypes and chzM, have representatives in the 400 genome collections, but a large part of the strains displays a different *cps* locus so they were classified as non-typable (*n*=140) (see Table S1).

### Genome diversity

A total of 23,261 families of genes was identified in the set of 400 genomes of *S. suis* using PPanGGOLiN. Among these families, 1,043 genes were persistent, representing 4.4% of the total. For the variable part of the pangenome, 6,489 shell genes, i.e. gene families present at intermediate frequencies, and 15,729 cloud genes, i.e. families present at low frequencies, were identified. This corresponds to 27.9% and 67.6% of the total families of genes, respectively. Alignment of the persistent genes was used to build the phylogenetic tree shown in Fig. S1. Strains can be grouped in seven major clades with two divergent clades: clades 6 and 7 (used as outgroup to root the tree). The 65 strains belonging to the divergent clades exhibit only 76–88% ANI with the other genomes.

The high diversity of the 400 genomes collection was also illustrated by the MLST typing results since 194 strains exhibit a particular sequence type (ST) and 206 strains display a novel ST (Table S1). STs previously described as associated with a pathogenic pathotype, i.e. ST1, ST17, ST25 and ST373 are all included in clade 1 (Fig. S2).

Genome sizes range from 1.9 to 2.6 Mb with a median value of 2.2 Mb (IQR=2.15–2.35 Mb) (Table S1). Analysis per clade indicated that most of the genomes with a high genome size are found in clade 5, whereas a large proportion of genomes of clades 1 and 2 have a smaller size. This corresponds to 87% of the >Q3 group and 70% of the <Q1 groups, respectively (Fig. S2 and Table S1). Strains with ST1 and ST17 mentioned above are in the group of smaller size genomes.

### Distribution of putative virulence genes

**Fig. 1.**
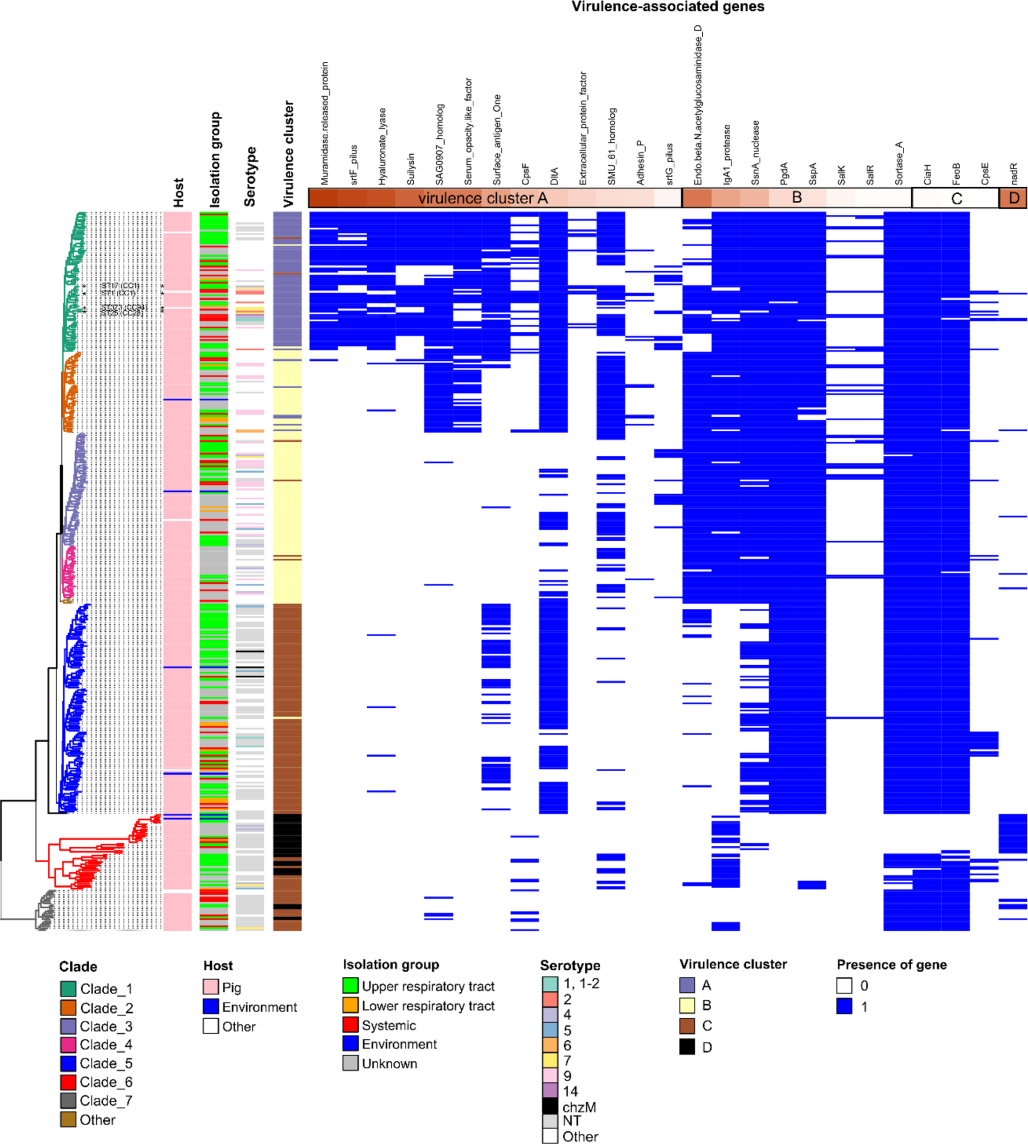
Characteristics of the 400 selected strains of *S. suis*. Genomes have been grouped in clades according to their phylogenetic distance determined by alignment of the persisting genes (see the ‘Methods’ section) as indicated by a phylogenetic tree at the left. STs previously described as associated with the pathogenic pathotype are indicated on the phylogenetic tree. Strains were also categorized according to their (i) host, (ii) isolation group, (iii) serotype, (iv) virulence cluster and (v) virulence-gene pattern. The virulence cluster of the strain was obtained by HCPC analysis (see the ‘Methods’ section). The grouping of the strains in four virulence clusters was made according to the presence (in blue) or absence (in white) of the most discriminant 25 virulence-associated genes (ordered by descending discriminatory power) among the 70 genes analysed. The colour code of clades and of other categories is shown at the bottom of the figure.

The 400 genomes were screened for the presence of 70 virulence-associated markers described in *S. suis*. MCA coupled with HCPC analysis enabled the separation of genomes in 4 clusters according to the presence/absence of the 25 most discriminatory virulence markers (Fig. 1, Table S1). The most discriminatory markers of the first cluster (virulence cluster A) are muramidase release protein (MRP), SrtF pilus, hyaluronate lyase and suilysin, whereas endo-beta-*N*-acetylglucosaminidase is strongly associated with the second cluster (cluster B on Fig. 1). Most of the strains of the other clusters do not harbour these virulence markers. The NadR marker is strongly associated with cluster D of virulence, which is found only in strains of the divergent clades 6 and 7 (Fig. 1).

Clusters A and B are characterized by a higher number of putative colonization/virulence factors (*χ*²=430.09, *P*=5.0×10^−4^) with 76.3% and 22.7% of the genomes of these clusters belonging to the ‘>Q3 virulence group’ (with more than 52 virulence markers), respectively. Among the 90 genomes with a low number of virulence markers (‘<Q1 virulence group’, with 34–45 virulence markers), 57.8% are grouped in cluster C and 40.0% in cluster D. This fits with phylogenetic clades (*χ*²=923.91, *P*=5.0×10^−4^, Fig. 1) with 93.6% of strains of clade 1 grouped in cluster A of virulence, clades 2 to 4 mostly found in cluster B (86.7% and 93.1% respectively) and clade 5 in cluster C (99.1% of the strains) (Fig. 1). This is less obvious for the divergent clades 6 and 7 that appear in two clusters (C and D with 26.2–73.8% and 78.3–21.7% repartition, respectively). There is however no statistical link between these virulence clusters and the sampling site (‘isolation-group’) of the strains (*χ*²=12.84, *P*=0.13>0.05), nor between the sampling site and the number of virulence markers (*χ*²=8.83, *P*=0.32>0.05). This is illustrated by 14 strains of the systemic group having a low number of virulence markers and 42 strains isolated from the upper respiratory tract having a high number of virulence markers. The 11 strains that harbour an extracellular protein factor (EPF), all strains except one that have a muramidase-released protein (MRP, *n*=70) and all strains except two with a suilysin (*n*=55) belong to phylogenetic clade 1. However, only one strain with the *epf+ mrp+ sly*+ genotype has been isolated from a systemic infection, whereas five strains with this genotype were isolated from the upper respiratory tract of a pig.

When spotting the genome size versus the number of virulence genes per genome, it appears that genomes with the highest number of virulence genes (>52) have a smaller size (Fig. S3). This corresponds to clades 1 and 2 reported above as having a high proportion of small genomes. As shown in Fig. 1, clade 1 includes strains with an ST previously associated with a pathogenic pathotype (ST1 and ST17 from CC1, ST25 from CC28 and ST373 from CC94 [54]) and all the strains with serotype 2.

### Diversity and distribution of AMR determinants

**Fig. 2.**
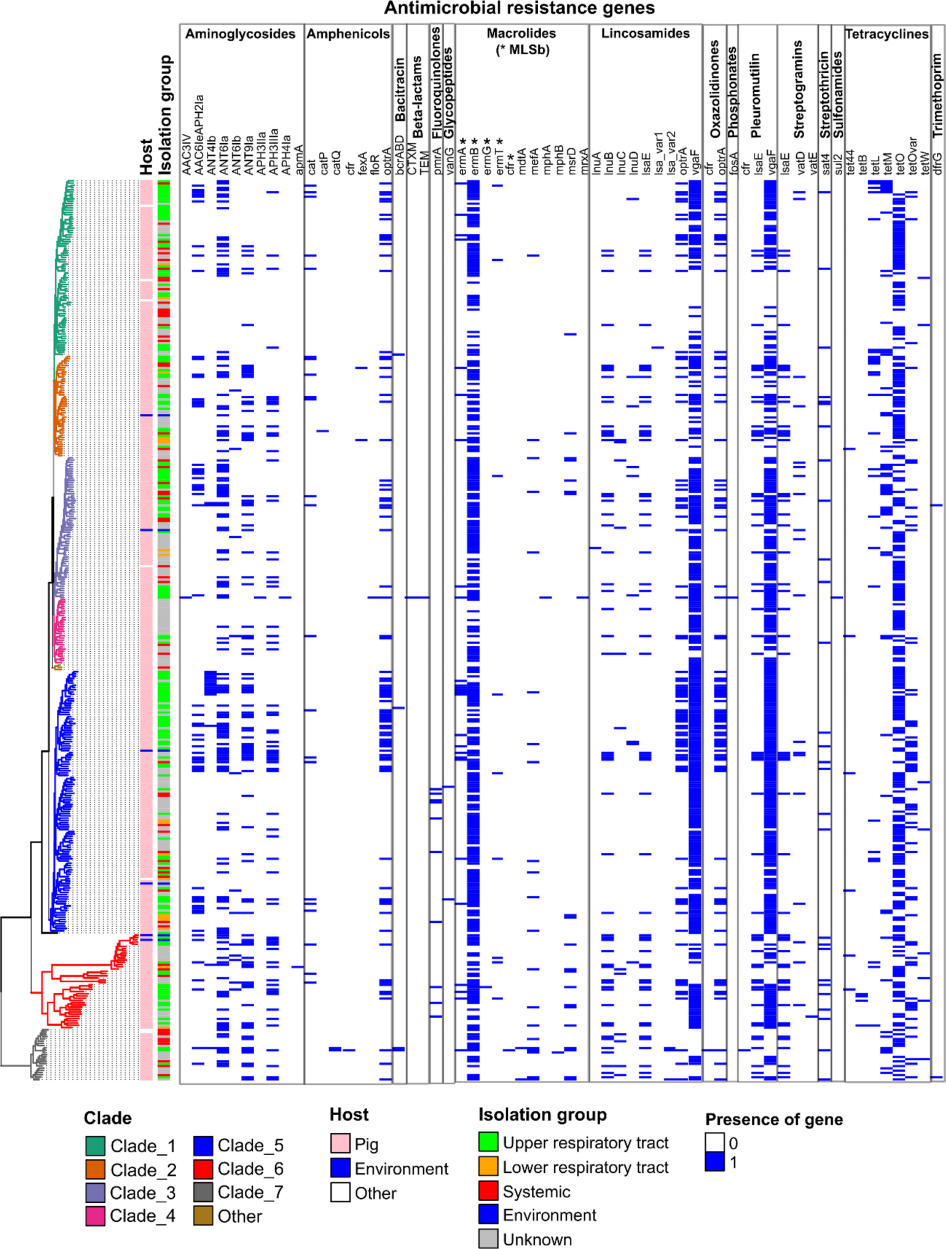
AMR gene pattern in the 400 genomes of *S. suis* according to the clade, host and isolation group of the strains. AMR genes have been classified per class of antibiotics targeted by the resistance determinant. Some AMR genes confer resistance to multiple antibiotics and thus appear several times on the diagram. Genomes have been grouped in clades according to their phylogenetic distance determined by alignment of the persisting genes as indicated by the phylogenetic tree at the left. The colour code of clades and of other categories is shown at the bottom of the figure.

A total of 1,754 AMR genes, corresponding to 53 different kinds of AMR determinants and expected to confer resistance to 12 classes of antibiotics, were detected in the 400 genomes of *S. suis* (Table S1, Fig. 2). Strains harbour between 1 and 16 AMR genes, but this number varies according to the isolation site of the strain. Strains isolated from the upper respiratory tract have a significantly higher number of AMR genes than strains from systemic infections, with a median number of AMR genes of 6 and 3, respectively (*χ*²=30.7, *P*=1×10^−3^, Fig. S4). The AMR genes are also not evenly distributed among phylogenetic groups, with strains of clade 6 having more AMR genes than strains of clades 1 and 4 (*χ*²=54.0, *P*=5×10^4^, Fig. S5).

The most prevalent AMR genes are *vga*(F) (*n*=289), *erm*(B) (*n*=285) and *tet*(O) (*n*=190) with a combination of these 3 genes in 58 strains (Figs 2 and S6). Other AMR genes were scarcer. *cfr*, which confers resistance to multiple antibiotics including amphenicols and macrolides, was detected in one strain belonging to the divergent cluster 7 (strain SFJ44). This strain also carries the *catQ* AMR determinant that encodes a chloramphenicol acetyltransferase. The *catQ* gene is found in another strain of the divergent cluster 7 (strain SC2B29-1). Another gene encoding a chloramphenicol acetyltransferase, *catP*, was detected in one *S. suis* strain (DAT300). The *fex(A*) gene that encodes an efflux protein of the major facilitator superfamily conferring resistance to amphenicols was detected in two strains of clade 2. *floR*, which also mediates resistance to amphenicols but through efflux by a specific transporter, was detected in a single *S. suis* strain of serotype 4 isolated from a pig in China (strain 1367). Strain 1367 also carries CTX-M-65 and TEM-1-b genes that encode an extended-spectrum beta-lactamase and are surrounded by copies of Insertion sequences (IS) of the IS26 family, *fos*A3 that encodes a Mn^2+^ and K^+^-dependent glutathione S-transferase that inactivates fosfomycin (phosphonate antibiotic) and *sul2* that confers resistance to sulfonamides. Strain 1367 likely harbours a multiresistance cluster that includes six AMR genes (CTX-M-65, *fosA3*, *floR*, *sul2,* APH(4)-Ia and AAC(3)-IV). This cluster of AMR genes shares multiple almost identical (99–100% of nt identity) regions with the pESI-like plasmid p423_13 described in a strain of *Salmonella* Infantis isolated in Brazil [55] (Fig. S7). However, many assembly gaps due to the presence of multiple ISs do not enable to draw definite conclusions either about the location (chromosome or plasmid) or the clustering of the AMR genes in *S. suis* 1367. This strain harbours the highest number of AMR genes: 16 in total including the previously mentioned multiresistance cluster and TEM-1b and additional ANT(6)-Ia, APH(3′)-IIa, *erm*(A), *erm*(B), *mphA*, *mrxA*, *optrA*, *tet*(O) and *vga*(F) genes. The *mph*(A) and *mrx*(A) genes are located in the same operon and encode a macrolide-inactivating enzyme (2′-phosphotransferase I that phosphorylates 14- and 15-membered ring macrolides) and an efflux pump, respectively. *mdt*(A), found in two strains isolated from a pig in China (SC2B29-1 and HCJ31), and *pmrA* (detected in ten strains) also encode an efflux pump. *vat*(E) that encodes a streptogramin A acetyltransferase was detected in strain 784_18B on a contig of 1,144 bp that ends with a 15 bp sequence identical to ISSu10. In the same strain, the contig that contains the *erm*(B) gene also includes a sequence of ISSu10 (transposase gene). A linkage between *vat*(E) and *erm*(B) is thus possible in this strain. Another infrequent AMR gene is *tet*(B) that encodes an efflux pump for tetracyclines. It was found in four strains, all belonging to the divergent cluster 6.

### Analysis of ICEs and IMEs

#### Distribution of ICEs and IMEs in the analysed genomes

Among the 400 genomes studied, 566 ICEs and 1,035 IMEs were detected (Table S1 and Fig. S8). ICEs and IMEs were detected in all the clades even in the two divergent ones, and a high diversity of MGE combinations was observed (Fig. S8). Only nine strains are devoid of ICEs and IMEs. The other strains host up to ten ICEs/IMEs. The number of MGEs varies according to the phylogenetic clade (*χ*²=90.1, *P*=5×10^−4^). Clades 1, 2 and 7 have more MGEs than the other clades (>30% of the strains included in the >Q3 group compared to 2% for clade 6), whereas clade 6 has less MGEs (71% in <Q1 group compared to 9–15% for clades 1, 2 and 7) (Figs S2 and S9). The number of ICEs, IMEs or total MGEs does not differ significantly between serotypes or isolation sites (*P*>0.05).

#### Diversity of ICEs and IMEs in the genomes

**Fig. 3.**
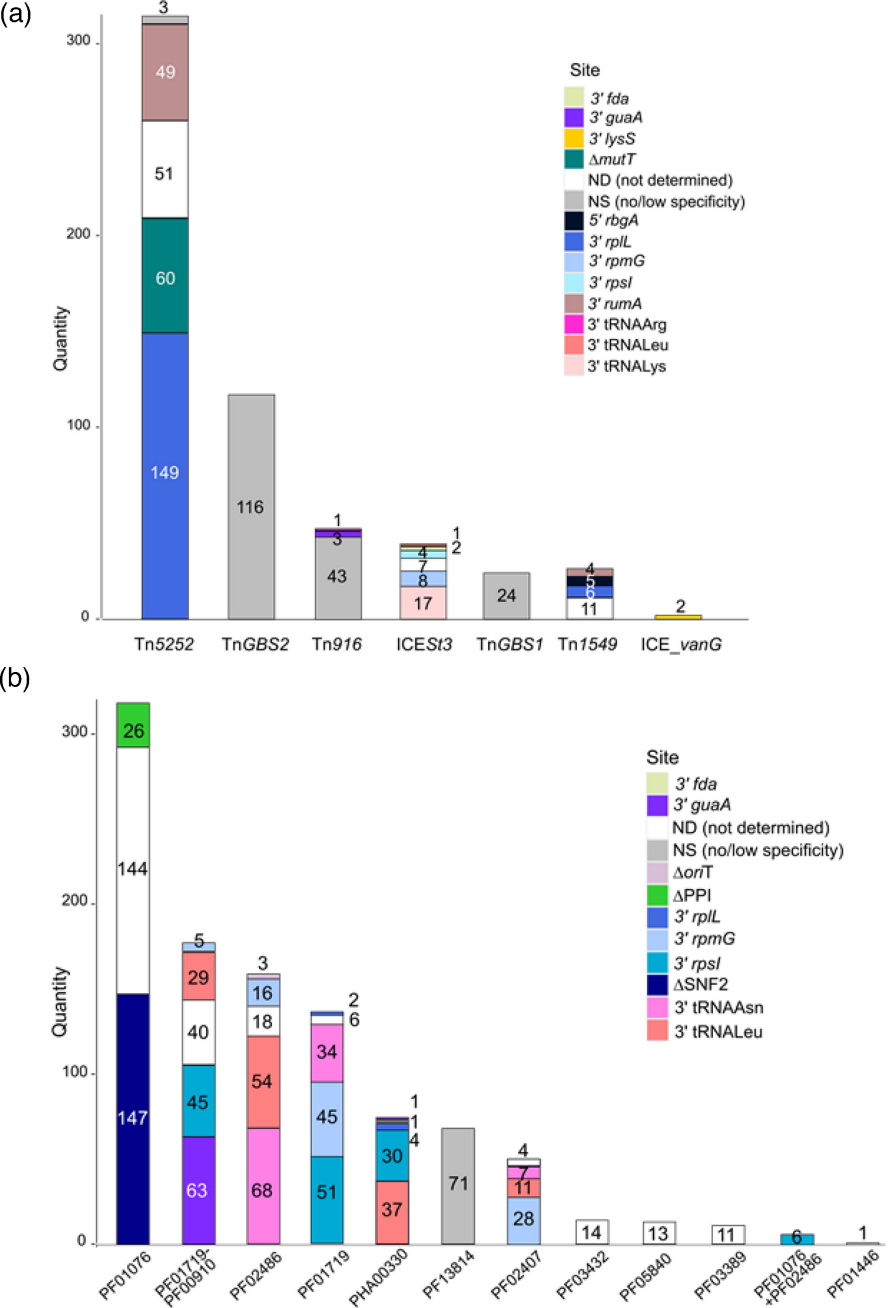
Stacked histogram showing the families of (a) ICEs and (b) IMEs detected in the 400 genomes of *S. suis* and their integration sites (shown in different colours, see legend on the right). When the gene is interrupted by the integration of the element, it is mentioned by a delta symbol before the name of the target gene.

ICEs found in the 400 genomes belong to 3 superfamilies and 7 families according to their conjugation module (as described in other streptococci [56]): (i) the Tn*5252* superfamily, which includes the following families of ICEs: Tn*5252* (*n*=312), Tn*GBS2* (*n*=116), Tn*1549* (*n*=26) and ICE_*vanG* (*n*=2) families; (ii) the Tn*916* superfamily that includes ICEs of the Tn*916* (*n*=47) and ICE*St3* (*n*=39) families; and (iii) the Tn*GBS1* family (*n*=24) (Fig. 3a). The seven families of ICEs are present in all the clades even in the two divergent ones (Fig. S8). Twelve different specific integration sites were observed (Figs 3a and S8). As already reported [57,58], some of these ICEs (Tn*916 sensu stricto*, Tn*GBS2* and Tn*GBS1*) have a low specificity of integration and target AT-rich regions. More surprisingly, three ICEs of the Tn*5252* family use a transposase with a DDE motif (DDE transposase) DDE transposase for their integration and excision, which likely results in less specific integration than other ICEs of the same family (indicated as ‘ICE_*Tn5252_NS*’ in Table S1). These three ICEs share closely related conjugation modules, with relaxases, VirB4 and CP identities ranging from 96.5 to 97.9, 99.2 to 99.5 and 97.0 to 97.8% respectively. However, they are only distantly related to other ICEs of the Tn*5252* family, as evidenced by the low relaxase sequence identity (42.4–45.3%) compared to other members of the Tn*5252* family.

One strain (SC1B9) isolated from a pig in China seems to host four different Tn5252 ICEs (Table S1). The genome of this strain is incomplete and the presence of multiple ICEs with similar conjugation modules impairs genome assembly, so it is not possible to determine if these ICEs all have a full conjugation module. We also identified two strains (strains 1782 and LSS81) that likely carry a tandem of ICEs of the Tn*5252* family at the *rplL* integration site. In addition, one strain (TMW_SS088) hosts two Tn*916*.

We found multiple examples of mosaic elements. Tn*916* nested in an ICE of the Tn*5252* family was identified in ten genomes. In addition, many ICEs of the Tn*5252*, Tn*GBS2* and ICE_*vanG* families host one or several group II introns. Group II introns are MGEs that consist of a catalytically active RNA, called ‘ribozyme’, which can self-splice from the chromosome and an intron-encoded protein (IEP) that helps splicing of the intron and stabilizes the ribozyme structure for reverse splicing into DNA during intron mobility [59]. Two ICEs of the Tn*5252* family found in strain GD-0088 both host three group II introns. Three other group II introns were also discovered in this strain (two in an ICE of the *vanG* family and one in an intergenic region). Target genes are *virB4* and the gene encoding the helicase of the ICEs (with two different integration sites). Comparison of the aa sequence of the IEPs of these introns indicated that they can be divided into three families (sharing 21–41% of identity). Each family is characterized by a specific integration site (corresponding to a DPEXE motif in the PF01935 domain of VirB4, a DNMLXI motif in the PF00176 domain or a WRPXDXX motif in the PF00271 domain of the helicase). A fourth family of group II introns that targets the gene encoding the CP of a Tn*5252* ICE was identified in strain LSS99. This group II intron targets a DEFAN motif in the PF02534 domain of the CP and has an IEP with only 22% of identity with the IEP of the three other families. blastx search using the IEP sequences of the four families of group II introns led to 212 hits for the 400 genomes of *S. suis*.

IMEs are classified according to the family of their relaxase [25]. Twelve different families or combinations of relaxases were identified in the 400 genomes of *S. suis*. The most prevalent family of IMEs harbours a relaxase with a PF01076 domain (MobV family, *n*=317). Most relaxases can be associated with a range of integrases that determines their specificity (or low specificity) of integration. Ten specific target genes were identified (Fig. 3, Table S1). The most frequently targeted gene is the putative helicase gene (SNF2) of ICEs of the Tn*5252* family (*n*=147). New combinations of relaxase-integrase were discovered in particular for relaxases with a PHA00330 domain (found in three new integration sites: *fda*, *guaA* and *rplL*) and a PF05840 domain (integrated in *guaA*).

#### Co-occurrence of ICEs and IMEs

**Fig. 4.**
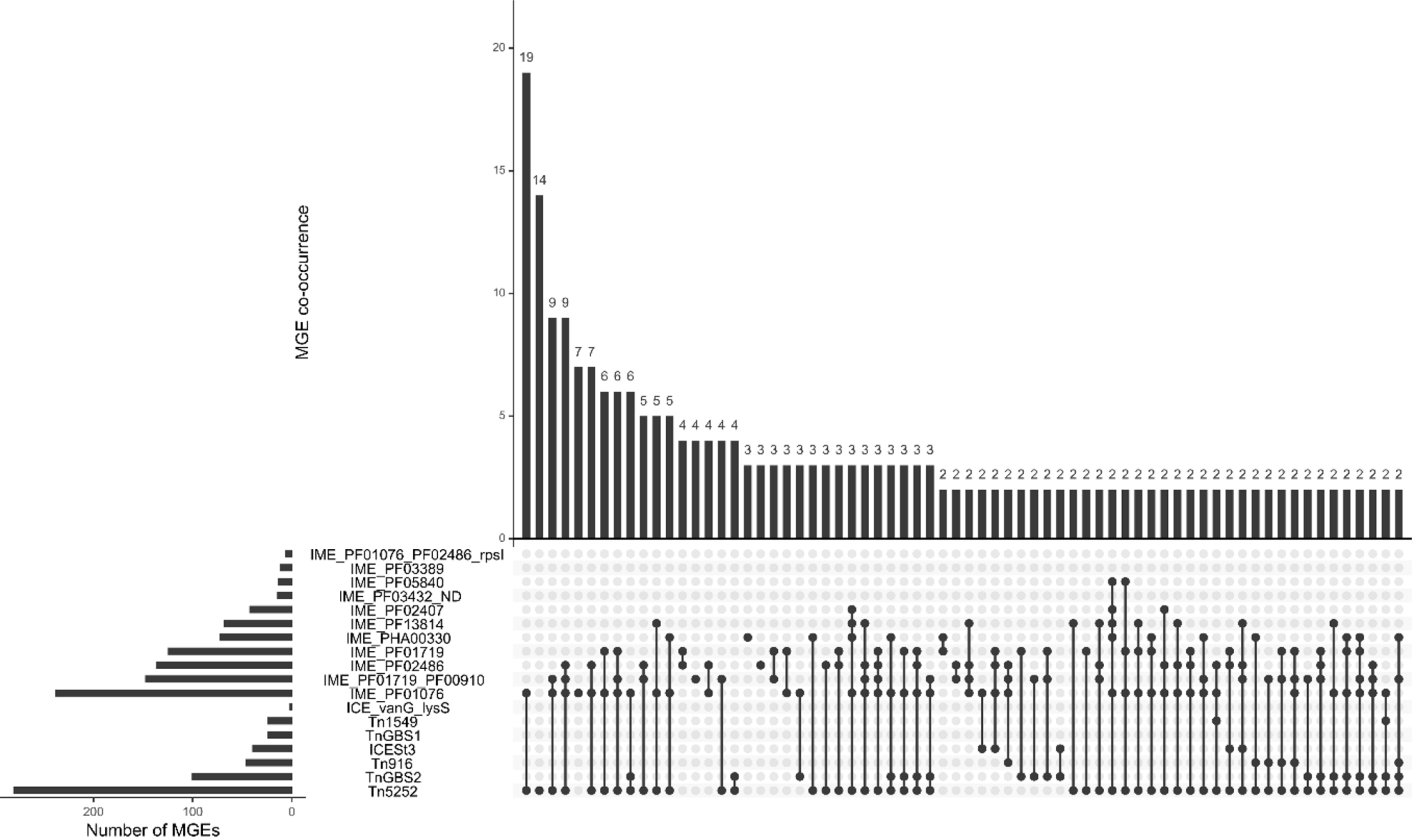
Co-occurrences of ICEs and IMEs identified in the 400 genomes of *S. suis*. The number of occurrences of each integrative element is indicated on the left with horizontal bars and the number of element co-occurrences by vertical histograms (with indication of the number of representatives on the top of the histogram). Combinations of elements are indicated in the centre of the figure by a circle in front of the elements in association, linked by a vertical line. Only those encountered at least twice are indicated on the figure.

Since IMEs with a MobV relaxase (with a PF01076 domain) integrated in the putative helicase gene (SNF2) of Tn*5252* ICEs are the most prevalent, the IME_PF1076-Tn*5252* association is the most frequent ICE-IME combination observed (*n*=258, Fig. 4).

We also studied the association between ICEs and IMEs located outside an ICE in order to draw hypotheses about their possible trans-mobilization in other bacterial cells. Two preferential IME-ICE associations were identified: IME_PF01719-ICE*St3* and IME_PF13814-Tn*GBS2*. However, in both cases, the association is at the limit of statistical significance (*χ*²=3.9, *P*=0.04 and *χ*²=5.3, *P*=0.02 respectively). It should be pointed out that all the IMEs with a relaxase harbouring a PF01719 domain identified in this work (*n*=138) encode a CP of the same family (TcpA) as the CP of ICE*St3*. In addition, IMEs with a PF13814 relaxase (*n*=71) encode a CP that belongs to the same family (VirD4) as the CP of Tn*GBS2* ICEs.

#### ICEs and IMEs carrying AMR genes

To study the association between AMR genes and ICEs and IMEs, we filtered the AMR genes that were located within 60 kb of a relaxase gene (the maximal distance that was observed until now in ICEs found in *S. suis*). This was the case for 718 AMR genes (Table S2). No relaxase gene was found within a 60 kb distance for the 1,036 other AMR genes. However, many of them are located on short contigs that could not be positioned in the pseudochromosome obtained by alignment with reference complete genomes and were thus concatenated at the end of the pseudochromosome. For these AMR genes, it was not possible to study their association with ICEs or IMEs.

The most frequent associations were IME_PF01076 (with a MobV relaxase) with *tet*(O)-*erm*(B) genes (*n*=95) followed by IME_PF01076-*tet*(O) (*n*=56). These IMEs are themselves integrated in an ICE of the Tn*5252* family, inside a putative helicase gene or a peptidyl-prolyl-isomerase gene. Other AMR genes (ANT(4′)-Ib, ANT(6)-Ia, ANT(9)-Ia, APH(3′)-IIIa, *catP*, *erm*(A), *lnu*(B), *tet*(40), and *tet*(L)) were found on IMEs with a MobV relaxase (Fig. S10). Some of these IMEs were located inside an ICE (Tn*5252* or Tn*1549*). For example, in strain DAT300 of *S. suis*, the *catP* gene is located on an IME with a MobV relaxase that is integrated in the putative helicase gene of a Tn*1549* ICE (Fig. S10). Other IMEs with a MobV relaxase were integrated in a CDS encoding a putative HTH_regulator or less specifically for an IME with a DDE transposase (Fig. S10).

ICEs of the Tn*5252* family can carry not only *tet*(O)(-*erm*(B)) genes located on a nested IME but also many other AMR genes: AAC(6′)-Ie-APH(2″)-Ia, ANT(6)-Ia, ANT(9)-Ia, APH(3′)-IIIa, *erm*(A), *fexA*, *optr*(A), *lnu*(B), *lnu*(C), *lsa*(E), *sat*4, *tet*(O/32/0), *tet*(O/W/32/O) and *vat*(D) (Fig. S11).

Some other ICE/IME-AMR gene associations involving elements of other families were also detected: ICE-vanG_*lysS* and *lnu*(C)-*tet*(O)-*tet*(40)-ANT(6)-Ia, ICE*St3-cfr* or ICE*St3-bcrABD*, Tn*916-tet*(44), Tn*916-lnu*(D)-*vatD* and IME_MobT-*dfrG*.

### Competence genes

A total of 341 out of the 400 strains analysed (85%) harbour the whole set of genes required for the acquisition of extracellular DNA by transformation (Tables S1 and S3 and Fig. S12). All of these strains, except one (strain 126), show a pherotype I. The genes that are the most frequently inactivated (through an early stop in most of the cases) are the ComEC component of the DNA transport system (*n*=22), the oligopeptide permease (Opp) for ComS import (*n*=11), the ComX sigma factor (*n*=11) and components of the ComY pilus (*n*=7) (Fig. S12). The proportion of non-competent strains is higher in the divergent clade 7 than in other clades (52% compared with 3–24% for the other clades, *χ*²=46.5, *P*=5×10^4^).

### Analysis of DSs

#### RM systems and orphan methylases

RM systems were searched by two methods: by doing blast analysis against the REBASE Gold database and using the PADLOC tool (see the ‘Methods’ section). A total of 2,035 RM systems were identified by the blast method: 756 of type I, 648 of type II, 236 of type III and 395 of type IV systems and 992 orphan methylases (Table S1). With PADLOC, 542 type I, 625 type II, 226 type III and 37 type IV were detected in the 400 genomes of *S. suis* (Table S1). Two-thirds of the strains (*n*=267) have 3 different categories of RM systems and 118 strains harbour the 4 categories of RM systems (Fig. S13). Most isolates fell within a 3–7 range for the number of RM systems, but 57 genomes had more (>Q3 group with 8–14 RM systems, Table S1).

**Fig. 5.**
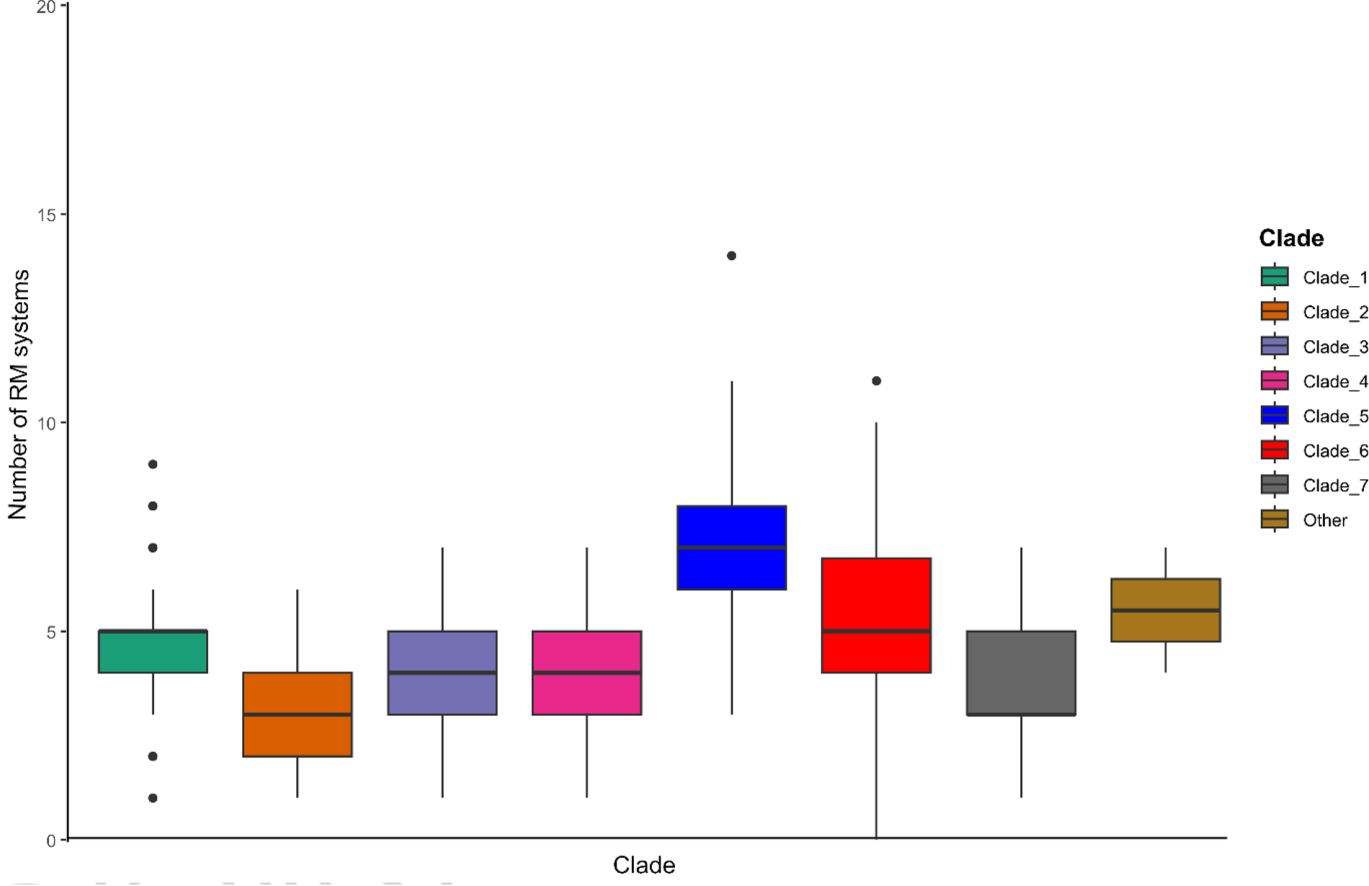
Number of RM systems according to the clade of the strains. The box plots represent the data distribution with the IQR between the upper and lower quartiles, Q3 (75th percentile of the data dispersion) and Q1 (25th percentile of the data). The median values (middle 50% of the data) are indicated by a horizontal line inside the boxes. Outliers are indicated as black circles. The boxes are coloured differently according to the phylogenetic clade (see legend on the right).

We analysed if there is a link between a high RM content (i.e. belonging to the >Q3 group) and other features of the strains. The number of RM systems differs according to the phylogenetic clade with more RM systems in clade 5 and less RM systems in clade 2 (*χ*²=128.0, *P*=5×10^−4^). The proportion of strains of clade 5 having a high RM content reaches 38% compared with 0–21% for the other clades. For clade 2, 35% of the strains belong to the <Q1 group compared with 0–22% for the other clades (Fig. 5).

Manual analysis of the blast results against the REBASE Gold database enabled the identification of methylase genes not associated with other RM subunits. These genes were categorized as orphan methylase genes (Table S1). Numerous orphan methylases were identified in the genomes (*n*=992) with up to eight orphan methylase genes per genome. Deeper analysis indicated that three orphan methylases (M.Lla5598I, M. LmoJ3I and M. Cdi630II) were frequently found in the genomes (*n*=496, 113 and 99, respectively) (Fig. S14). We analysed the position of these orphan methylase genes on the chromosome to see if they were located in the vicinity (60 kb upstream or downstream) of a relaxase gene. We found that at least 368 orphan methylase genes (37%) were located close to a relaxase gene and thus could be located on an ICE or an IME. Further analysis indicated that most of them correspond to M.La5598I (*n*=297) that encodes a C5-cytosine methyltransferase with an unknown recognition site located on a Tn*5252* putative ICE. Two ICEs of the Tn*1549* family carry an orphan methylase gene: M2.HpyAVIP, a C5-cytosine methyltransferase that recognizes a CCTC motif, and M.Cdi630III, a C4-cytosine methyltransferase that recognizes a CCSSGG motif. In addition, five categories of IMEs carrying an orphan methylase were discovered. Two distantly related IMEs with a PF13814 relaxase encode an N6-adenine methyltransferase that targets a RGATCY motif. IMEs with a PF01719 relaxase, integrated in the *rpsI* site, encode M.SmoLIV, an N6-adenine methyltransferase that targets a CGWAG motif. IMEs with a PF01076-MobV relaxase harbour M.Hpy99XI, a C5-cytosine methyltransferase that recognizes an ACGT motif. Finally, IMEs with a PF03389-MobQ relaxase encode M.Bce14579I, a C5-cytosine methyltransferase that recognizes a GCSGC motif.

#### CRISPR-Cas systems

**Fig. 6.**
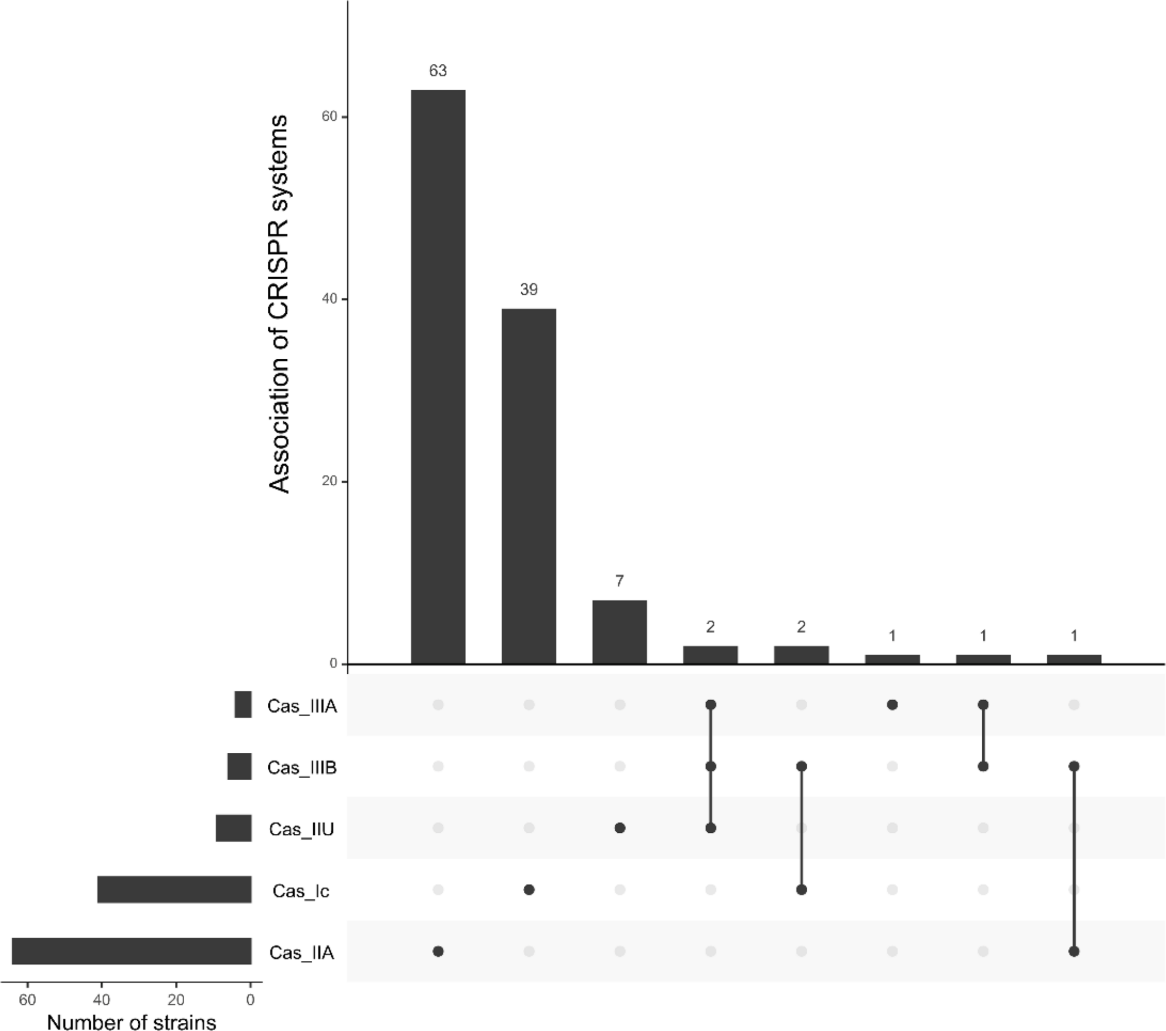
Occurrence and associations of various categories of CRISPR systems found in the 400 genomes of *S. suis*. The number of occurrences of each CRISPR-Cas system is indicated on the left with horizontal bars and the number of system associations by vertical histograms (with indication of the number of representatives on the top of the histogram). Combinations of CRISPR-Cas systems are indicated in the centre of the figure by a circle in front of the systems in association, linked by a vertical line.

CRISPR-Cas systems were searched by three methods: (i) by doing blast analysis using the sequences of CRISPR-Cas systems already described in *S. suis* (CRISPR1-IIU, CRISPR2-Ic and CRISPR3-IIa found in strains 8830, NCTC10446 and 6407, respectively) [33], (ii) using CRISPR-Cas finder [50] and (iii) using PADLOC [49]. A total of 113 CRISPR systems were retrieved by blast analysis (see column ‘CRISPR-Cas’ in Table S1). These systems were also identified by the CRISPR-Cas finder tool, which additionally detected ten more systems that belong to the Cas-IIIa (*n*=4) and Cas-IIIb (*n*=6) families (Table S1). The two most abundant CRISPR-Cas systems were Cas-IIa (*n*=64) and Cas-Ic (*n*=41) systems (Fig. 6, Table S1). Six strains carried more than one CRISPR-Cas system (Fig. 6, Table S1). These strains all belong to the divergent clade 6. Analysis of the genomes with PADLOC revealed almost the same number of CRISPR systems (only one system was missed, Table S1).

We examined the location of CRISPR-Cas systems in order to see if some are carried by MGEs. We found only one example of CRISPR array (but not associated with Cas genes) located on an IME with a PF13814 relaxase in strain IMT40738. This array contains two repeats and one spacer. We also identified two MGEs [with an integrase but no relaxase that we will call mobile genetic islands (MGIs)] carrying a CRISPR array with several spacers.

The CRISPR-Cas finder tool also gave as output the number and position of spacers in the CRISPR arrays associated with Cas genes. This number, which reflects the activity of the CRISPR systems [60], differed largely between systems (5 to 123 spacers). A significant inverse correlation (*χ*²=10.2, P=0.006) was observed between the number of these spacers and the number of AMR genes in the strains (chi-square test using the variable ‘Spacers_group’ to explain the variable ‘AMR_total_group’, see Table S1). This means that a higher number of spacers (>Q3 for the Spacers_group) were found in the group of strains that have less AMR genes (<Q1 group for AMR genes). A significant link was also found between this number of spacers and clades (*χ*²=70.1, *P*=5×10^−4^), with a higher number of spacers (>Q3 group) in clades 1 and 3. We analysed the sequence of these spacers using blastn and found a hit in databases for 1,396 sequences (35% of the total 3,939 sequences, Table S4). Most of the hits corresponded to phage sequences (*n*=1182, Fig. S15). A few genes of phages were particularly targeted (encoding holin, phage tail or portal proteins or terminase, Table S4). Other MGEs were less targeted by CRISPR-Cas systems: MGIs (70 hits), plasmids (62 hits) and ICEs (defective or not, 43 hits). For ICEs, several hits correspond to genes encoding cell surface proteins or relaxases (Table S4). Self-targeting was also observed (39 hits against other chromosomal loci, Fig. S15).

#### Other DSs

**Fig. 7.**
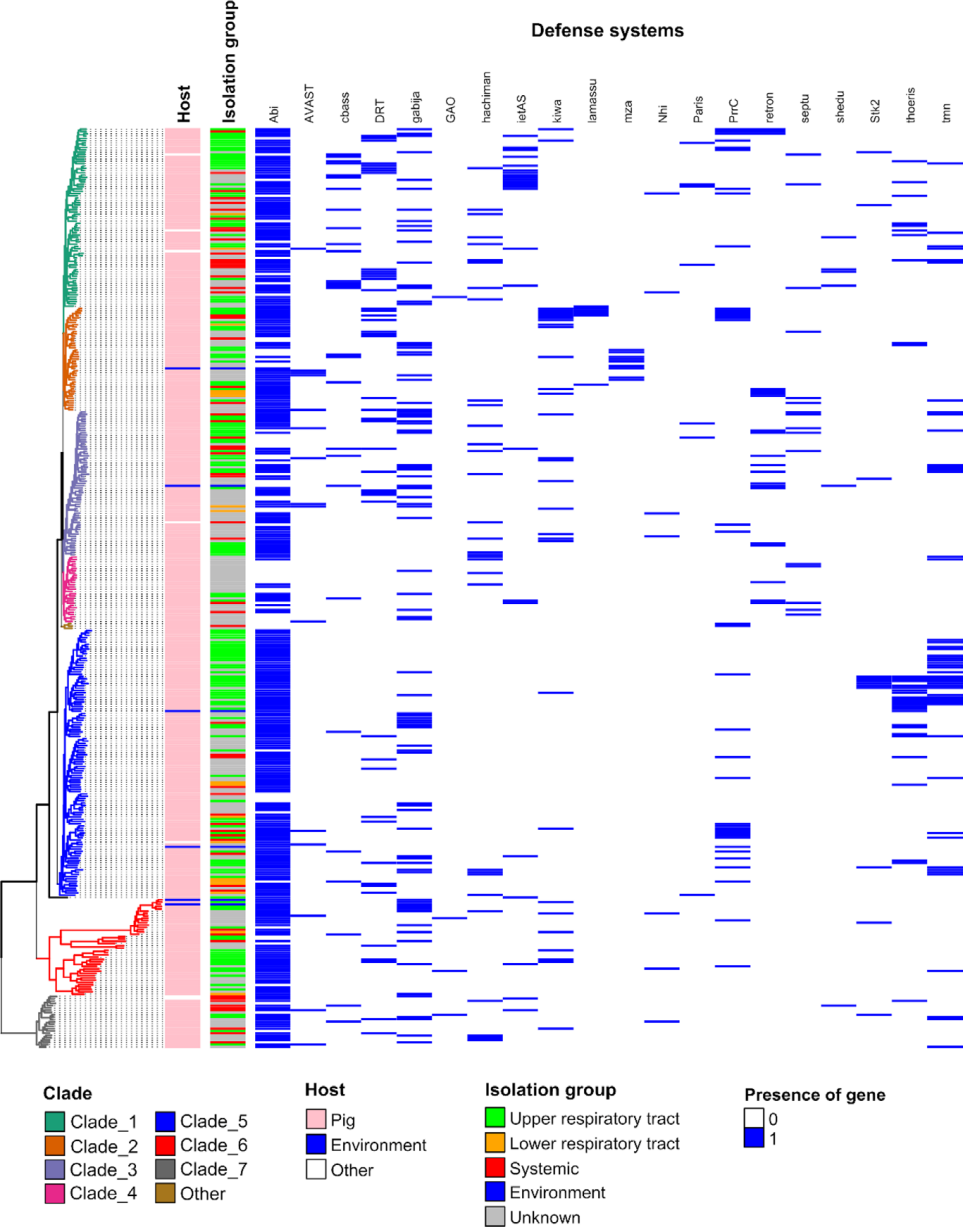
Pattern of DSs (excluding RM and CRISPR-Cas systems) in the 400 genomes of *S. suis* according to their phylogenetic clade, host and isolation site. Genomes have been grouped in clades according to their phylogenetic distance determined by alignment of the persisting genes as indicated by the phylogenetic tree at the left. The colour code of clades and of other categories is shown on the bottom of the figure.

Using PADLOC, we searched for other types of DSs. A total of 1,074 DSs other than RM systems and CRISPR-Cas systems were detected. Twenty different types of DSs were detected: Abi, AVAST, CBASS, DRT, Gabija, GAO, Hachiman, ietAS, Kiwa, Lamassu, mza, Nhi, Paris, PrrC, retron, septu, shedu, Stk2, Thoeris and Tmn (Table S1, Fig. 7). The repertoire of DSs was diverse across all isolation groups (8 to 20 types) and clades (with a maximum of 9 DSs in clade 4 to a maximum of 20 types in clade 1) but also inside clades. Abi systems were the most abundant DSs since most of the strains (*n*=325) harbour at least one Abi. Some strains have up to 6 representatives of this category of DSs (579 Abi systems detected in total, Table S1). Gabija and DRT systems that also target phages were also frequently found (in 84 and 45 strains for a total of 88 and 49 systems, respectively) whatever the isolation site of the strain (Fig. 7). Another system targeting phages, Thoeris (*n*=34), was found mostly in strains of clades 1 and 5. By comparison, Lamassu and mza systems were found only in strains of clade 2 (Fig. 7).

When including RM systems and CRISPR-Cas systems described above, the whole defence armoury of *S. suis* can be very impressive (up to 17 DSs detected per genome). The number of DSs depends on the phylogenetic clade (*χ*²=74.9, *P*=5×10^−4^), with a higher abundance of DSs (>Q3 group) in clades 5 and 6 and less DSs in clades 2 and 4 (Fig. S16). Interestingly, strains without ICE (cluster called ‘5ICE_0’) exhibited a lower number of DSs (*χ*²=39.2, *P*=5×10^−4^).

## Discussion

Despite the heterogeneity of the *S. suis* species, PPanGGOLiN showed a good performance for analysing the pangenome of the 400 genomes included in this study. The number of persistent genes obtained (1,043) for the 400 genomes analysed is close to the 1,164 core genes described by Shelyakin *et al*. [61] when studying the pangenome of *S. suis* using 25 genomes. The high proportion (more than 20% of the total genes) of shell genes, i.e. gene families present at intermediate frequencies in the species, is in agreement with the description of an open pangenome for this bacterial species and its ability to adapt to a changing environment [61].

By HCPC, 4 clusters of strains could be discriminated according to their pattern of 25 virulence-associated factors. These clusters fit with the phylogenetic clades obtained by alignment of the core genes. MRP, SrtF pilus, hyaluronate lyase, suilysin (SLY), endo-beta-*N*-acetylglucosaminidase and NadR were the most discriminant virulence markers among the 70 genes analysed. Hyaluronate lyase and NadR were already identified as discriminatory factors in our previous work conducted on a collection of French isolates of *S. suis* [18]. The *epf+ mrp+ sly*+ genotype is extensively used to predict the virulence potential of *S. suis* [5,62]. Five strains of *S. suis* isolated from the upper respiratory tract of pigs show this genotype. This suggests that even strains isolated from the upper respiratory tract of pigs could have a high invasive potential.

A large proportion of strains of clade 1, which show the highest number of virulence genes, have a genome of smaller size than average. This is the case of the ST1 strain included in the study, in accordance with a previous work that pointed out a genome reduction occurring in this lineage [14]. Association between bacterial pathogenicity and smaller genomes and fewer genes has been broadly documented in other species [63].

A huge amount of AMR genes (1,754 genes) was detected in the 400 genomes of *S. suis*. Most of them have already been described in this bacterial species and were shown to confer resistance to 12 families of antibiotics: aminoglycosides, amphenicols, bacitracin, glycopeptides, macrolides, lincosamides, oxazolidinones, pleuromutilin, streptogramins, streptothricin, tetracyclines and trimethoprim [17,18, 20, 21, 62, 64, 65]. However, 11 AMR genes were detected for the first time in *S. suis* to our knowledge: *catP*, CTX-M-65, *floR*, *fosA*, *mdt*(A), *mph*(A), *mrx*(A), *pmrA*, *sul2*, TEM-1-b and *vat*(E). CTX-M-65, *floR*, *fosA*, *mph*(A), *mrx*(A), *sul2* and TEM-1-b were detected in the same strain (strain 1367). This strain with serotype 4 belongs to clade 3 and harbours 16 AMR genes in total (the 7 genes mentioned above and AAC(3)-IV, ANT(6)-Ia, APH(3′)-IIa, APH(4)-Ia, *erm*(A), *erm*(B), *optrA*, *tet*(O) and *vga*(F)). It would be interesting to determine the resistance phenotype of this strain. CTX-M-65 and TEM-1-b confer resistance to penicillin, aztreonam and first-, second- and third-generation cephalosporins in many Gram-negative pathogens (*Escherichia coli*, *Klebsiella pneumoniae*, *Acinetobacter baumannii*, *Pseudomonas aeruginosa* and *Enterobacter* spp.) [66]. In strain 1367 of *S. suis*, these genes are surrounded by copies of IS of the IS26 family that likely contributed to their integration in the *S. suis* genome. *floR* is a dominant driving factor of florfenicol resistance in Gram-negative bacteria such as *Enterobacteriaceae*, *Pasteurellaceae* (in particular in *Glaesserella parasuis* and *Actinobacillus indolicus* that are pig pathogens), *Flavobacteriaceae* and *Lysobacteraceae* [67,68]. In these species, the *floR* gene is mainly located on plasmids [67,68]. In *Salmonella enterica* serovar Infantis, this gene is included in a multiresistance gene cluster located on a megaplasmid called pESI-like [69,71]. *fosA3* is the most commonly reported plasmid-mediated fosfomycin resistance gene among *Enterobacteriaceae*. It was likely mobilized from the chromosome of *Kluyvera georgiana* (belonging to *Enterobacteriaceae* but generally considered as non-pathogenic) by an IS26-mediated event [72]. All these examples point to a major role of ISs in the dissemination of AMR genes between bacterial species both in Gram-negative and Gram-positive bacteria. The CTX-M-65, *fosA3*, *floR*, *sul2*, APH(4)-Ia and AAC(3)-IV genes found in strain 1367 could be located on a multiresistance cluster that shows similarity to the pESI-like megaplasmid described in *Salmonella* Infantis [55]. In strain 1367 of *S. suis*, *mph*(A) and *mrx*(A) are in operon with *mphR*(A) that encodes a putative transcriptional regulator. In *E. coli*, the transcription of the *mph*(A)-*mrx*(A)-*mphR*(A) operon is negatively regulated by the binding of the repressor protein, MphR(A), to the promoter of the *mph*(A) gene. The inhibition is released in the presence of erythromycin, leading to an inducible resistance to macrolides [73]. In *S. suis*, the high GC content of these genes (66% compared with 41% for the core genome of *S. suis* 1367) could prevent their expression as described in another low-GC Gram-positive bacterium, *Staphylococcus aureus* [74]. The Mdt(A) pump was reported to decrease strain susceptibility to macrolides, lincosamides, streptogramins and tetracyclines in *E. coli* but also in the Gram-positive bacterium *Lactococcus lactis* [75]. Pmr(A) was described in *Streptococcus pneumoniae* and confers resistance to fluoroquinolones [76]. The streptogramin A acetyltransferase Vat(E) was first described in *Enterococcus faecalis* and *Enterococcus faecium* [77,78]. In *E. faecium*, *vat*(E) is frequently linked to the *erm*(B) gene [78]. It could also be the case in *S. suis* since the contigs carrying these genes in strain 784_18B end by an IS of the same family. Strains of *S. suis* isolated from the upper respiratory tract included in this work have a significantly higher number of AMR genes than strains from systemic infections. This was already observed in a collection of French isolates of *S. suis* [18].

An exhaustive search of ICEs and IMEs was conducted in the set of 400 genomes of *S. suis*. Novel combinations of recombination–conjugation genes were discovered: ICEs with a Tn*5252* conjugation module with a DDE transposase, IMEs with a PHA00330 domain-relaxase integrated in *fda*, *guaA* or *rplL* and an IME with a PF05840 domain-relaxase integrated in *guaA*. This extends the known repertoire of *S. suis* chromosomal element transferring by conjugation. Several strains of *S. suis* carry multiple ICEs of the Tn*5252* or Tn*916* family, in tandem or not. This argues for an absence of immunity against ICEs of these families. Many cases of mosaic elements were also detected, which indicates the plasticity of the elements and their constant evolution. Group II introns targeting ICEs were found to be widespread and diverse in *S. suis*. Four families of group II introns were characterized, targeting specific sequences of ICEs of the Tn*5252*, Tn*GBS2* and ICE_*vanG* families. Interestingly, the motifs that are targeted by these group II introns are conserved between the four families of ICEs of the Tn*5252* superfamily (except for the helicase whose gene is absent in the Tn*GBS2* family), which explains why identical group II introns were found in ICEs of different families. Splicing of the intron and removal from its targeted sequences is required to allow the production of the full-length VirB4 and CP proteins that are essential for conjugative transfer of the ICEs. Compared to previous works [16,18], the high number and diversity of elements detected (566 ICEs and 1035 IMEs) provides a sufficient number of elements per family to study co-occurrences of ICEs and IMEs. Two preferential IME-ICE associations were identified: IME_PF01719-ICE*St3* and IME_PF13814-Tn*GBS2*. The PF01719 domain-relaxases belong to the Rep_2 protein family that includes rolling-circle replication (RCR) initiation proteins. The MobT relaxase of ICE*St3* harbours a PF02486 domain that is also found in RCR initiator proteins of the Rep-trans family involved in the maintenance of small plasmids of firmicutes [79]. In addition, IMEs with a relaxase harbouring a PF01719 domain encode a CP of the TcpA family. ICE*St3* also encodes a CP of this family, so the hypothesis of mobilization of IMEs with PF01719 relaxase_TcpA CP by this family of ICEs is conceivable. In the same way, IMEs with a PF13814 relaxase encode a CP that belongs to the same family (VirD4) as the CP of Tn*GBS2* ICEs, which is in favour of a possible mobilization of these IMEs by Tn*GBS2*.

We also studied AMR genes-MGE associations in the set of 400 genomes of *S. suis*. Most of the observed ICE-AMR associations have already been reported in *S. suis* [17,20, 21, 64, 80, 81]. The highly frequent *erm*(B)-*tet*(O) association reported in *S. suis* [4,17, 21, 82] is linked to the presence of IMEs with a PF01076 domain-relaxase carrying both AMR genes. Some other ICE/IME-AMR gene associations were detected for the first time in *S. suis*: Tn*5252-tet*(O/32/0), Tn*5252-vat*(D), ICE-*vanG*_*lysS* with *lnu*(C)-*tet*(O)-*tet*(40)-ANT(6)-Ia genes, ICE*St3-cfr*, ICE*St3-bcrABD*, Tn*916-tet*(44), Tn*916-lnu*(D)-*vatD*, IME_MobT-*dfrG*, IME_MobV-ANT(4′)-Ib, IME_MobV-*catP*, IME_MobV-*erm*(A), IME_MobV-*lnu*(B) and IME_MobV-*tet*(L). In many cases, the IMEs carrying AMR genes are integrated inside an ICE of the Tn*5252* or Tn*1549* families.

As observed previously for a collection of French isolates of *S. suis* [18], the majority of the strains harbour the full set of genes required for the acquisition of extracellular DNA by transformation. Zaccaria *et al*. [83] demonstrated that *S. suis* can reach a high level of natural competence with an efficient uptake mechanism. This suggests that natural transformation could be widespread in *S. suis* and could also contribute to horizontal transfer of AMR genes in addition to the conjugation mechanism. The components that are most frequently inactivated are the ComEC component of the DNA transport system, the oligopeptide permease (Opp) for ComS import, the ComX sigma factor and components of the ComY pilus. Okura *et al*. [33] also observed frequent inactivation of the ComEC and Opp components in *S. suis*.

A total of 2,035 RM systems, mostly of type I and type II, were identified in the 400 genomes of *S. suis* by blastp analysis against the REBASE Gold database. Most isolates have three to seven RM systems, which is higher than the average two RM systems reported for chromosomal replicons with a size similar to the *S. suis* chromosome (2.0–3.0 Mb range) by Oliveira *et al*. [84]. This indicates that RM systems are very abundant in *S. suis*. This could be linked to frequent HGT in this species [61], favouring the acquisition of RM systems. In addition, most of the strains of *S. suis* can acquire new genes by natural transformation, and Oliveira *et al*. [84] showed that RM systems are over-represented in naturally competent micro-organisms. A total of 992 orphan methylases were detected in this work, many of them being localized on an ICE or on an IME. ICEs and IMEs could thus frequently rely on an orphan methylase to escape degradation by RM systems when entering the host cell. Oliveira *et al*. [84] showed that most orphan methylases do not originate from a degradation of complete RM systems but were acquired as solitary components. It is thus possible that orphan methylases have other biological functions. Orphan MTases could act as partners to other, yet unidentified, system components involved in defence against invading bacteriophages. Defence mechanisms based on DNA methylation include not only RM systems but also the more recently discovered BREX systems [85]. In *E. coli*, a methyltransferase (PglX) acts as a toxin–antitoxin pair with an alkaline phosphatase (PglZ), and DNA methylation by PglX confers immunity [86]. In addition, DNA modifications protrude in the major groove of DNA and thus affect DNA interaction with proteins such as transcription factors or enzymes involved in the replication or repair of DNA [87]. Hence, orphan methylases could not only serve as antidotes against host RM systems but could also have a broader impact on host cell regulation or physiology [88]. By contrast with the work of Oliveira *et al*. [84], type I RM systems were found to be more abundant than type II RM systems in *S. suis* in our study. Among the prokaryotic genomes studied by these authors, 14 complete genomes of *S. suis* were analysed with one genome in common with our study. For this genome (corresponding to strain D12), three RM systems (two type II and one type IV) were detected by Oliveira *et al*. [84] compared to four in our analysis (two type II and one type IV but also a type I system). This system was confirmed by visual inspection of the genome. It is thus possible that other type I systems were missed in their study. For our blastp search of RM systems, we voluntarily did not set any identity cut-off to avoid missing components and preferred manual analysis to assemble components of RM systems. This proved to be fruitful because restriction subunits appear very distantly related to proteins of the database used (less than 30% of identity). This is the main explication of the discrepancy observed between results obtained using the blast approach and PADLOC for type I systems. PADLOC, whose very big advantage is to automatically combine RM components to identify putative RM systems, was also less efficient for the identification of type IV systems but was efficient in detecting type II and type III systems.

For CRISPR-Cas system detection, CRISPR-Cas finder supplanted the two other methods (blast and PADLOC) and enabled the detection of Cas-IIIa and Cas-IIIb systems not reported in *S. suis* until now. A few CRISPR arrays, not associated with Cas genes, were found located on MGEs: This could protect the MGE in case of transfer in a strain possessing a functional CRISPR-Cas system. Indeed, the CRISPR repeat units could be transcribed into small RNAs that mimic CRISPR RNAs and inhibit Cas proteins by forming non-productive effector complexes, thus serving as counter-defence against CRISPR systems [89]. Recruitment of only the CRISPR-array component could also provide a weapon against other MGEs and participate in inter-MGE competition [89]. Thanks to the CRISPR-Cas finder tool, we also analysed the number and sequence of the spacers included in the CRISPR arrays. A higher number of spacers were found in the group of strains that have less AMR genes. A similar observation was made for *E. faecalis*, with a significant inverse correlation between the presence of CRISPR-Cas system and the resistance to different antibiotics [90]. As expected, since phages are the most abundant MGEs and are harmful for bacteria by leading to cell lysis, most of the spacers corresponded to phage sequences. However, as previously described [91], self-targeting was also observed. It was proposed that, as in the case of the immune system in mammals, the prokaryotic adaptive system could have non-canonical functions beyond its protective action. Self-targeting by CRISPR-Cas systems could, by eliciting a DNA damage response, participate in genome evolution [92], particularly through the remodelling of genomic islands.

PADLOC enables the identification of an impressive amount of other DSs than RM and CRISPR systems in *S. suis*. More than 1,000 DSs, belonging to 20 different types of DSs, were detected. Most of them target phages. The whole defence armoury of *S. suis* can be very impressive, with up to 17 DSs detected per genome. Interestingly, strains without ICE exhibited a lower number of DSs. This could indicate that ICEs are spared by the bacterial DS arsenal that mainly targets phages and plasmids. The repertoire of DSs varies across isolation groups and clades. It also differs between closely related strains, as previously described in other bacterial species [93]. It was proposed to define a ‘pan-immune system’ by considering the diversity of DSs at the level of population rather than at the individual level. Such a pan-immune system could offer the versatility to combat a large panel of invading MGEs to ensure the survival of the bacterial population [93]. Restriction of HGT by DSs likely depends on various ecological and genetic factors, including the burden of MGEs and fitness effect of HGT in bacterial populations [94]. In addition, it was shown that DSs can act synergistically [29,92]. For example, the Abi response is not always bactericidal and can be bacteriostatic, enabling cell recovery once other DSs such as a RM system or a CRISPR system have cleared the infection [29]. Further work is needed to decipher these complex interactions between DSs.

The 65 strains belonging to two divergent clades included in this study exhibit only 76–88% ANI with the other genomes. This is very low compared with the standard 95% ANI threshold used to define a species [95]. Such diversity inside the *S. suis* species has already been described [13,15], but until now, no subspecies or novel species has been proposed to consider these divergent clades. A large proportion of the strains of these divergent clades harbours a particular pattern of virulence genes that is not found in the other clades. The divergent clade 6 also shows the highest number of AMR genes. In addition, both divergent harbour AMR genes not found in the other clades: *tet*(B) in four strains of clade 6, *cfr* and *catQ* in strains of clade 7. The six strains that harbour more than one CRISPR system all belong to the divergent clade 6. When considering all the DSs, clade six has a higher abundance of DSs than other clades. By contrast, these divergent clades do not differ from the other clades regarding their ICE-IME content, suggesting that extending HGT can occur between strains of these clades and clades of the core population.

## Conclusions

This collection of 400 high-quality genomes of *S. suis*, with its large geographical (11 countries), temporal (period of more than 20 years), host and isolation site scopes, is expected to be useful for other studies in *S. suis*.

Analysis of the pangenome of *S. suis* indicated a high proportion of accessory genes in agreement with the description of an open pangenome for this bacterial species and its ability to adapt to a changing environment.

Several virulence markers [MRP, SrtF pilus, hyaluronate lyase, suilysin (SLY), endo-beta-*N*-acetylglucosaminidase and NadR] well discriminate strains belonging to different phylogenetic clades. They could be useful for epidemiological purposes.

Two divergent clades that comprise 65 strains were highlighted in this work. Their very low ANIs compared with the genomes of the core population as well as their particular virulence gene pattern, AMR and DS contents support their grouping in novel sub-species or species even if they can exchange genes with *S. suis* by HGT.

Association between bacterial pathogenicity and smaller genomes holds true in the *S. suis* species. It could be a consequence of adaptation to the host and loss of unnecessary genes.

The highly diverse repertoire of AMR genes found in *S. suis* reinforces the idea that this species could serve as a reservoir of AMR genes for other species. A large proportion of them are carried by ICEs or IMEs, enabling their dissemination by conjugation. Natural transformation could also contribute to the dissemination of these AMR genes between *S. suis* strains, in particular when the AMR genes constitute a multiresistance cluster surrounded by ISs. Carriage strains of *S. suis* harbour a high number of AMR genes and need to be included in AMR surveillance. In addition, the presence of similar AMR genes in distant bacterial species argues for a global One Health approach for the surveillance of AMR dissemination.

Preferential associations between ICEs and IMEs were identified in this work. Experimental work is needed in order to see if such ICE helper-IME pairs are effective and more generally to see if trans-mobilization requires specific interactions between proteins of ICEs and IMEs. A retro-mobilization is also possible, i.e. a mobilization by an ICE present in the recipient cell and not in the donor cell that carries the IME. Other IMEs are integrated inside ICEs. It would be interesting to test experimentally whether their excision is required prior to mobilization by the ICE in another bacterial cell.

This work also shed light on parasitic overlooked elements, group II introns, that appear widespread and diverse in *S. suis* and complicate the identification of ICEs by leading to false pseudogenes for CP/VirB4 signature proteins of ICEs.

DSs appear very frequent and diverse in *S. suis*, even when comparing strains of the same phylogenetic clade. They could constitute a pan-immune system that ensures the survival of the bacterial population by providing weapons against a large panel of invading MGEs.

Self-targeting by CRISPR-Cas systems could, by eliciting a DNA damage response, participate in genome evolution of *S. suis*, particularly through the remodelling of genomic islands. This could notably contribute to the high plasticity and rapid evolution of ICEs. Two counter-defence mechanisms were identified on ICEs and IMEs: orphan methyltransferases and CRISPR array. Both mechanisms could also participate in inter-MGE competition. Orphan methylases could not only serve as antidotes against host RM systems but could also have a broader impact on host cell regulation or physiology in *S. suis*. In *S. suis*, defences appear directed mainly against phages, with chromosomal elements remaining mostly spared. Gain of beneficial genes, in particular AMR genes, that reside in MGEs offers the ability to adapt to different ecological niches and counter-balances the fitness cost of MGEs. We hypothesize that the close regulation of ICEs and IMEs that reduce their fitness cost, their frequent association with AMR genes and their counter-defences contributes to their ecological success in *S. suis*. More studies are needed in order to draw the full picture of MGE-MGE competition and host–MGE interactions.

## Supplementary material

10.1099/mgen.0.001521 Uncited Supplementary Material 1.

10.1099/mgen.0.001521 Uncited Supplementary Material 2.

10.1099/mgen.0.001521 Uncited Supplementary Material 3.

## Abbreviations

Abi: abortive infection
AMR: antimicrobial resistance
ANI: average nt identity
CBASS: cyclic-oligonucleotide-based antiphage signalling system
CDS: Coding DNA sequence
CP: coupling protein
DRT: defence-associated reverse transcriptase
DS: defence system
HCPC: hierarchical clustering on principal components
HGT: horizontal gene transfer
ICE: integrative and conjugative element
IEP: intron-encoded protein
IME: integrative and mobilizable element
IQR: interquartile range
MCA: multiple correspondence analysis
MGE: mobile genetic element
MGIs: mobile genetic islands
MLST: multilocus sequence typing
MRP: muramidase release protein
PCA: principal component analysis
RCR: rolling-circle replication
RM: restriction–modification
RT: reverse transcriptase
ST: sequence type
