# Supplementary material for: Pan-immune system, mobilome and resistome in Streptococcus suis
Source: Microb Genom. 2025 Sep 29;11(9):001521. doi: 10.1099/mgen.0.001521 (PMC12479173; doi:10.1099/mgen.0.001521)
Supplement: Uncited Supplementary Material 1. [file mgen-11-01521-s001.pdf]

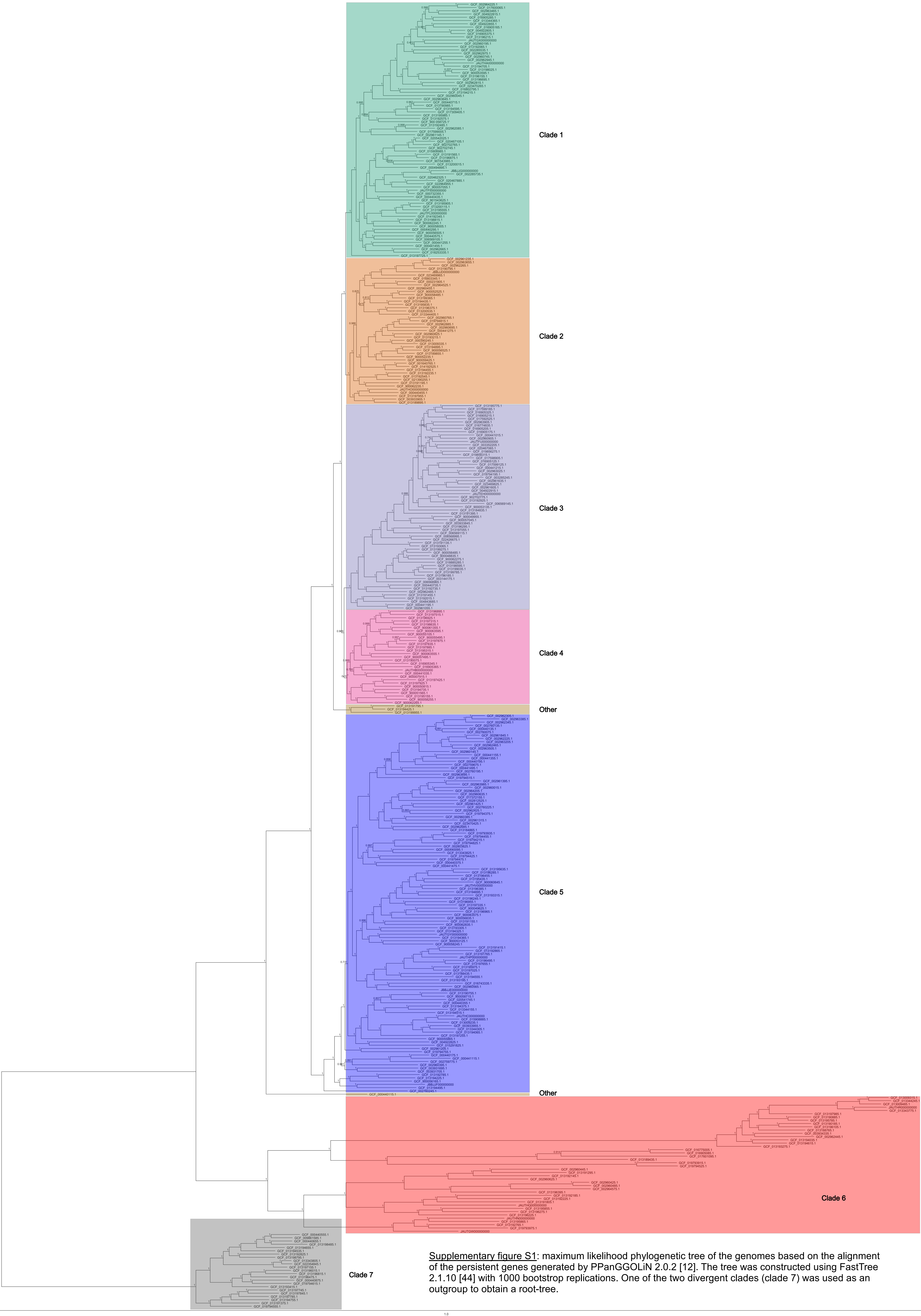

Supplementary figure S1: maximum likelihood phylogenetic tree of the genomes based on the alignment of the persistent genes generated by PPanGGOLiN 2.0.2 [12]. The tree was constructed using FastTree 2.1.10 [44] with 1000 bootstrop replications. One of the two divergent clades (clade 7) was used as an outgroup to obtain a root-tree.

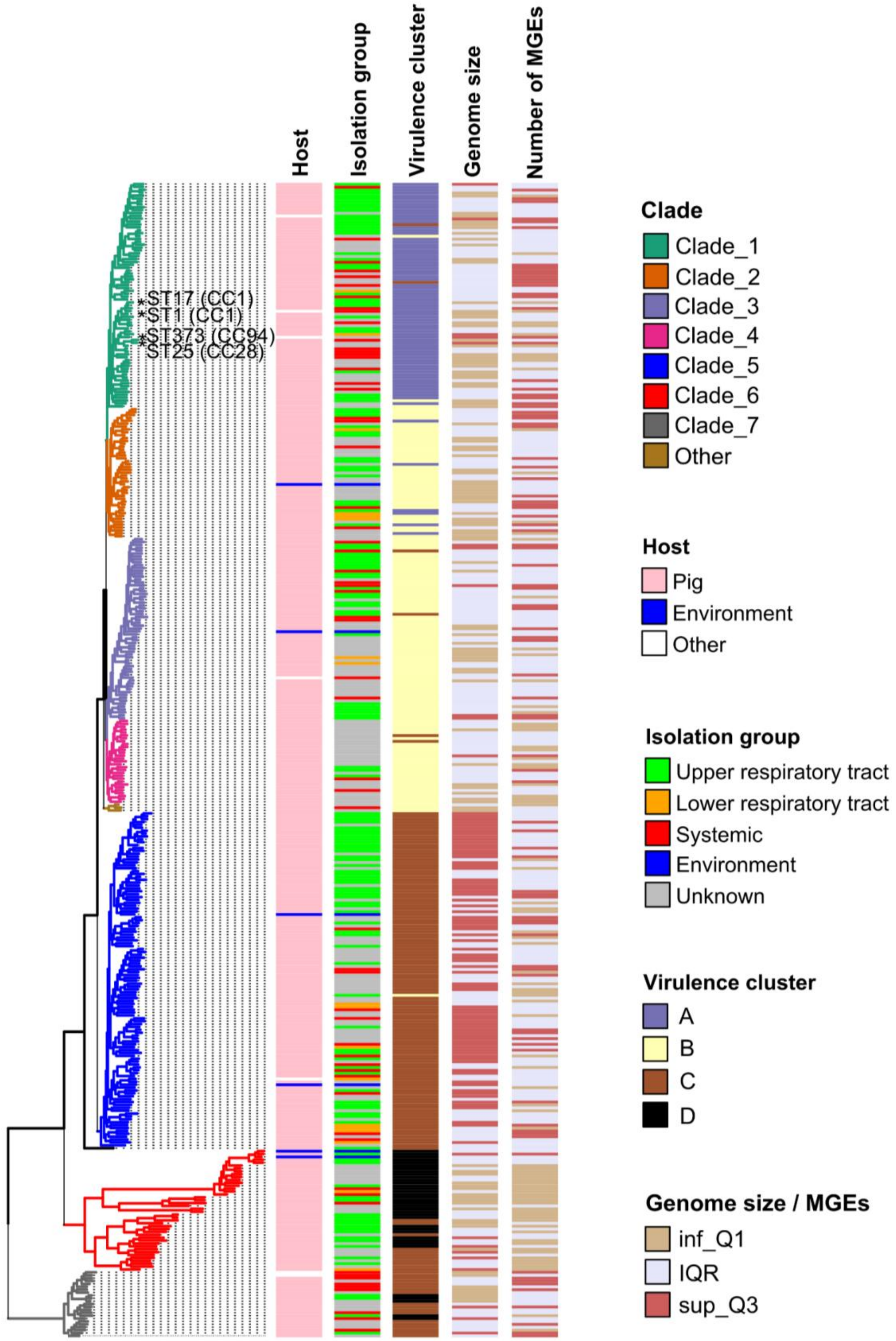

Figure S2: Genome size and number of Mobile Genetic Elements (MGEs) in the 400 *S. suis* strains according to their phylogenetic clade, host, isolation site and virulence cluster. Genomes have been grouped in clades according to their phylogenetic distance determined by alignment of the persisting genes as indicated by the phylogenetic tree at the left. The virulence clusters were obtained by Hierarchical Clustering on Principal Components on the basis of the presence or absence of the most discriminant 25 virulence-associated genes among the 70 genes analysed. The color code of clades and of other categories is shown on the right of the figure.

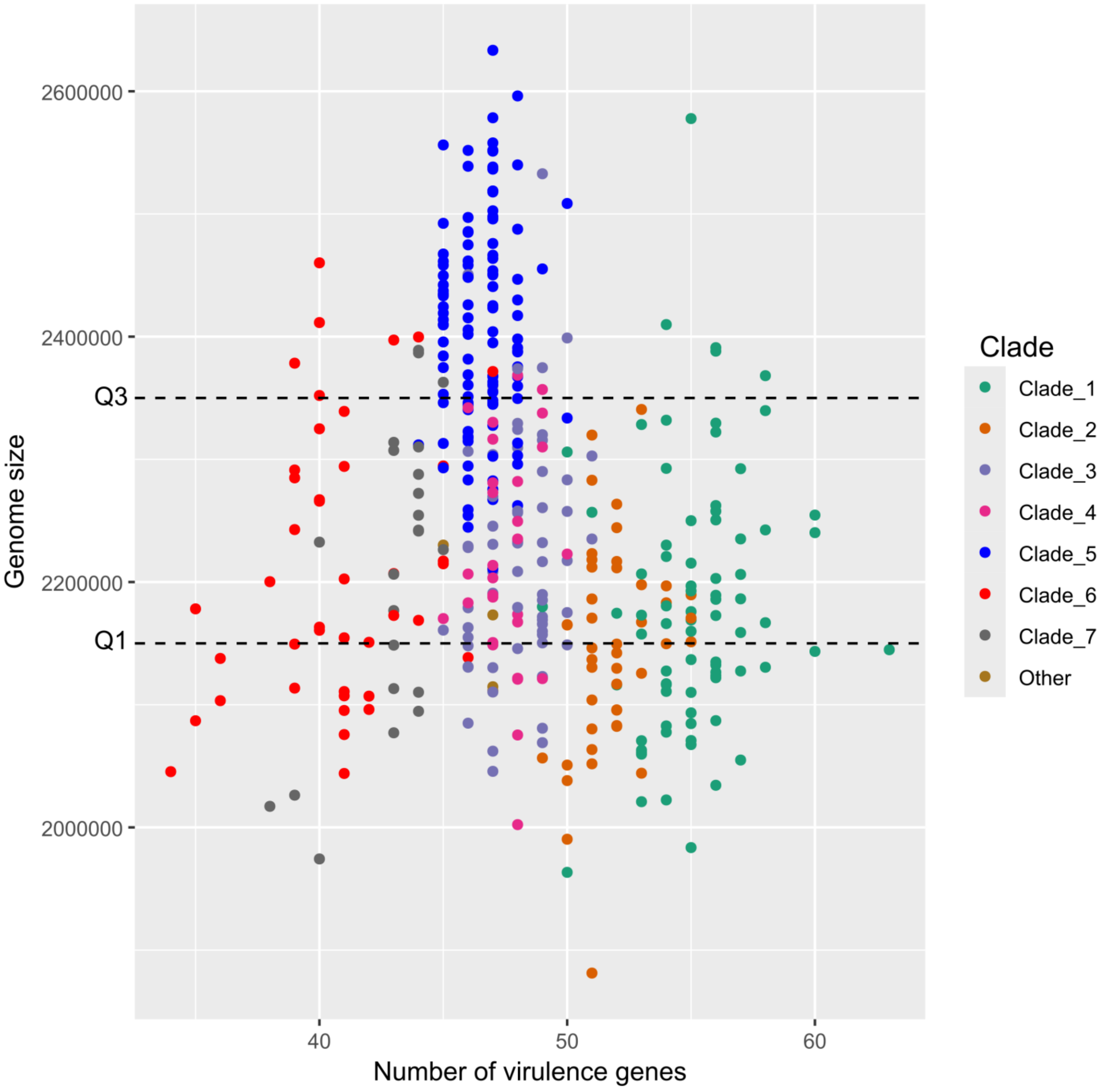

Figure S3: Scatter plot of the genome size of the 400 *S. suis* strains according to the number of total virulence genes detected in the genomes. The color of the circles indicated the phylogenetic clades (see legend on the right).

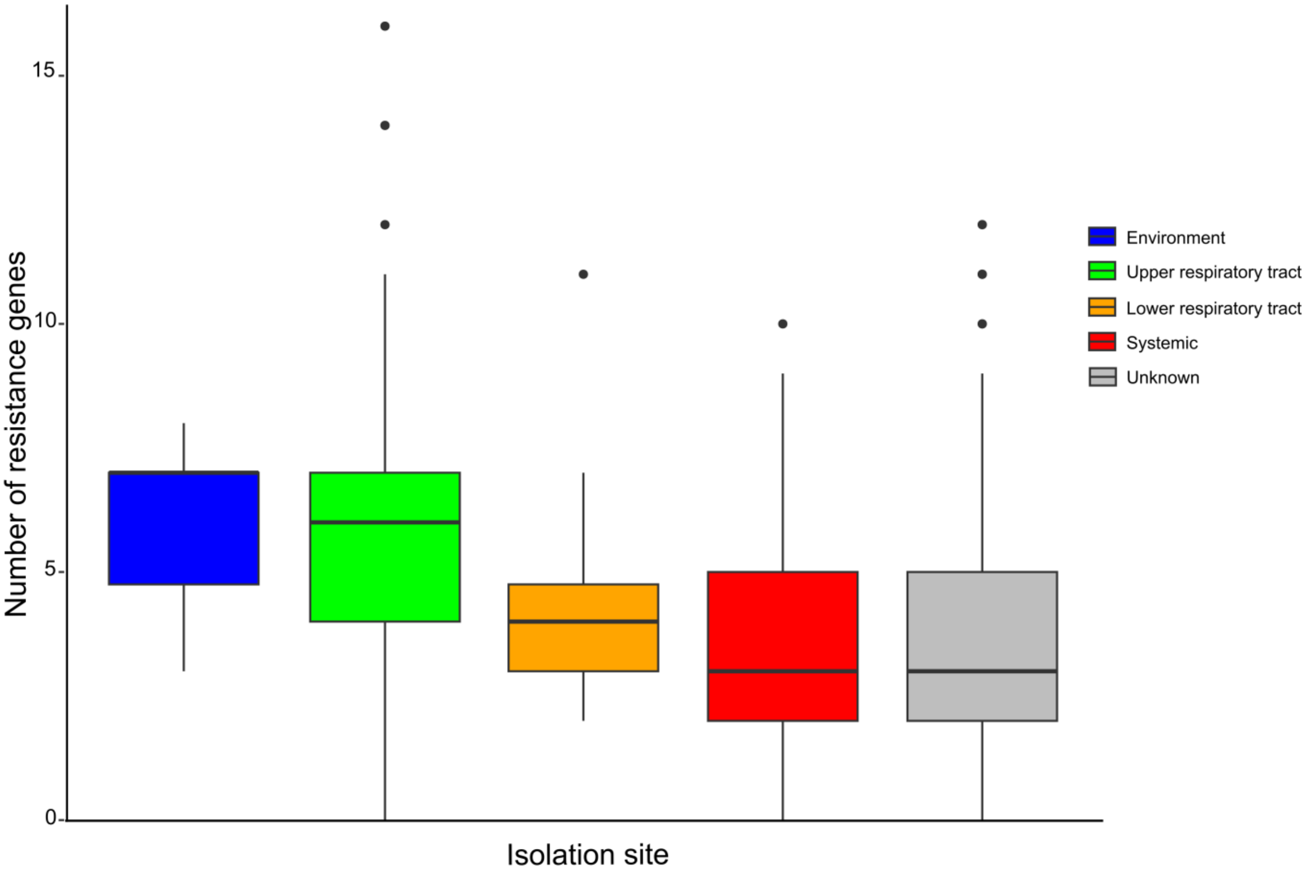

Figure S4: Number of antimicrobial resistance genes according to the isolation site of the strains. The box plots represent the data distribution with the interquartile range between the upper and lower quartiles, Q3 (75th percentile of the data) and Q1 (25th percentile of the data). The median values (middle 50% of the data dispersion) are indicated by a horizontal line inside the boxes. Outliers are indicated as black circles. The boxes are colored differently according to the isolation site (see legend on the right).

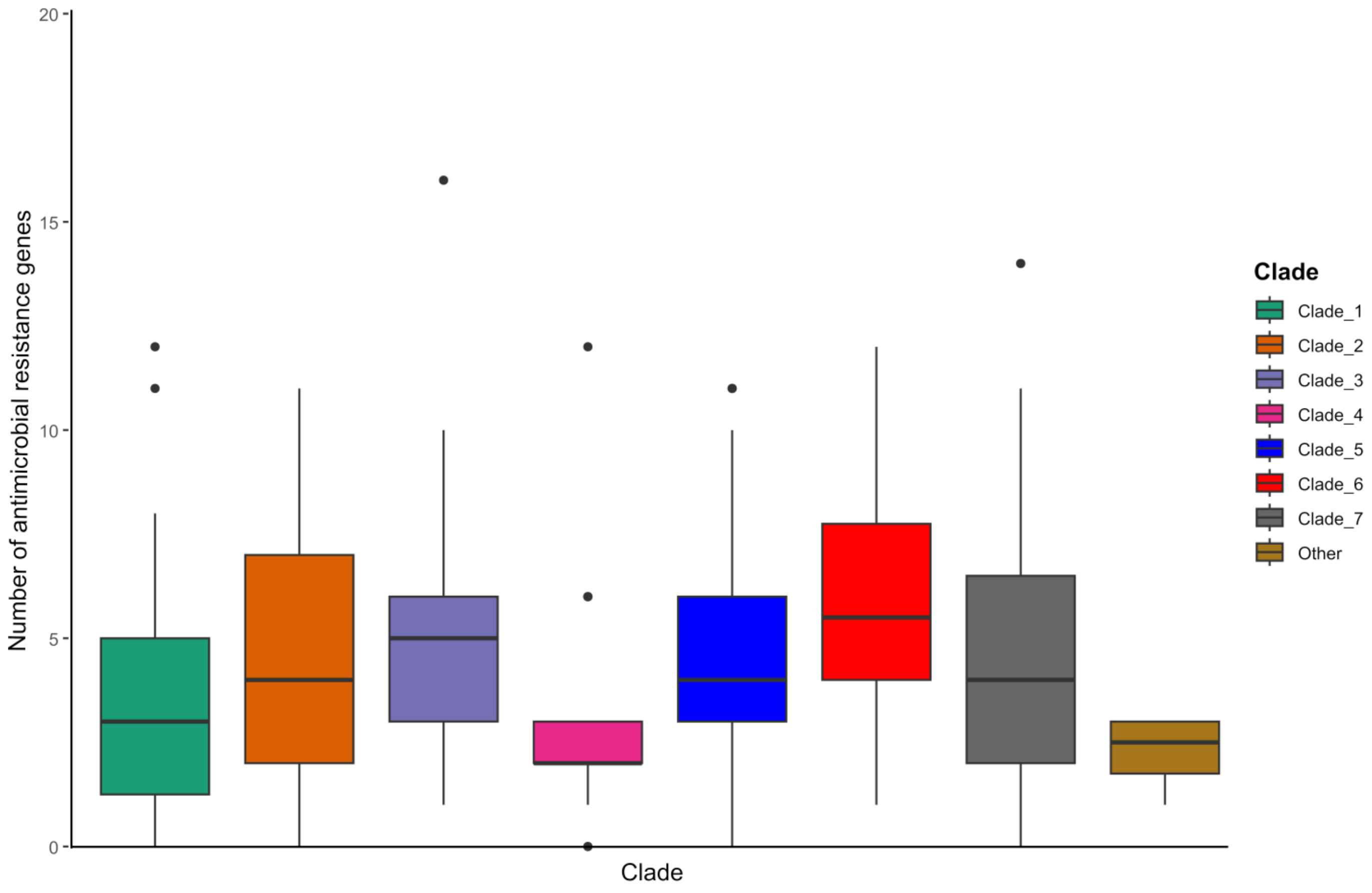

<u>Figure S5</u>: Number of antimicrobial resistance genes according to the clade of the strains. The box plots represent the data distribution with the interquartile range between the upper and lower quartiles, Q3 (75th percentile of the data dispersion) and Q1 (25th percentile of the data). The median values (middle 50% of the data) are indicated by a horizontal line inside the boxes. Outliers are indicated as black circles. The boxes are colored differently according to the phylogenetic clade (see legend on the right).

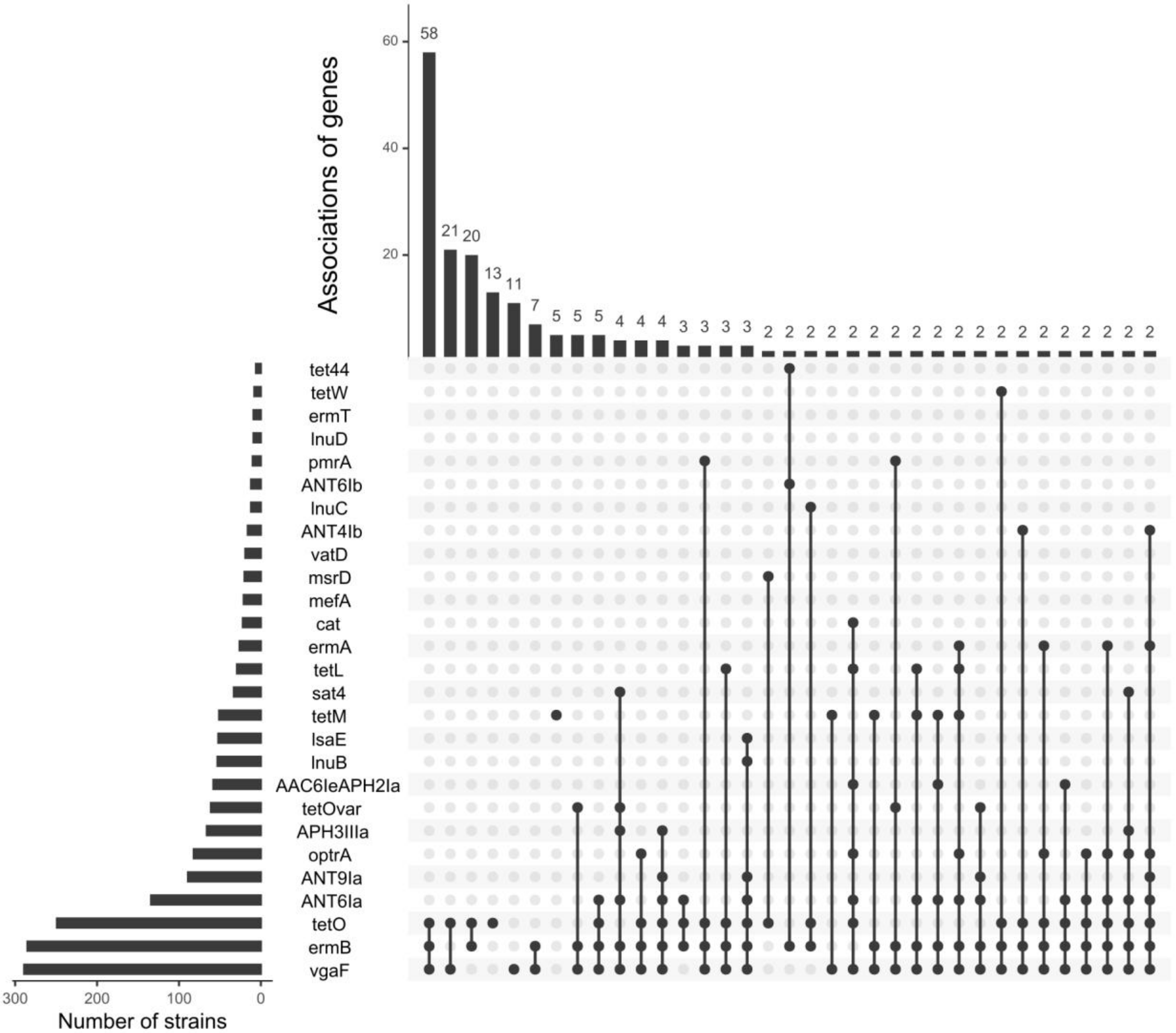

Figure S6: Occurrence and associations of antimicrobial resistance genes in the 400 genomes of *S. suis*. The number of occurrences of each resistance gene is indicated on the left with horizontal bars and the number of gene associations by vertical histograms (with indication of the number of representatives on the top of the histogram). Combinations of genes are indicated in the center of the figure by a circle in front of the resistance genes in association, linked by a vertical line.

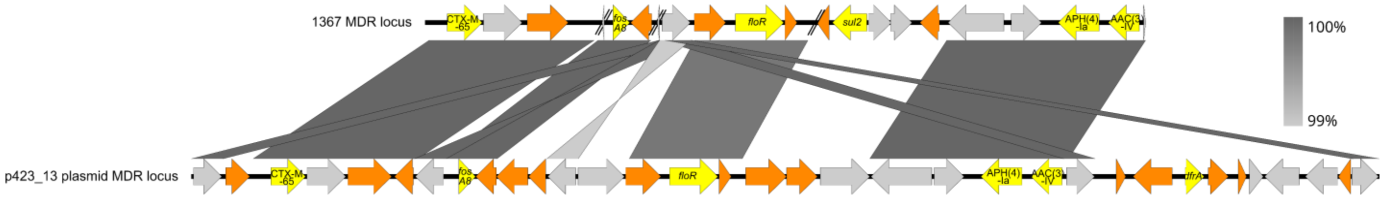

<u>Figure S7</u>: Comparison of the multidrug resistance locus found in strain 1367 of *S. suis* and the one found on plasmid p423_13 of *Salmonella* Infantis. Antimicrobial resistance genes are indicated as yellow arrows, transposase genes as grey arrows and other genes as arrows in orange. The regions of similarity are indicated in grey betwen the two sequences (with the corresponding scale on the right).

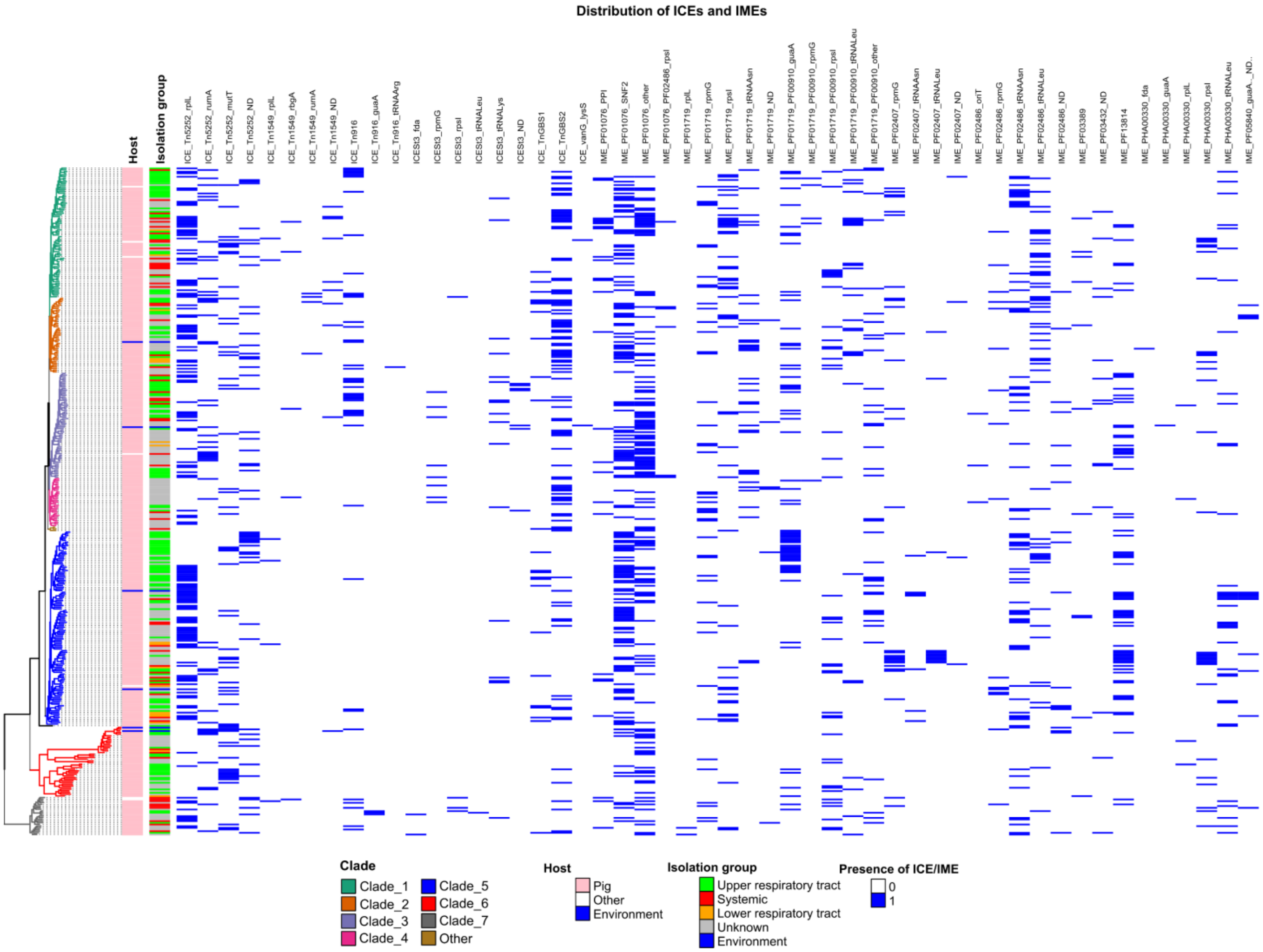

<u>Figure S8</u>: Distribution of Integrative and Conjugative Elements (ICEs) and Integrative Mobilizable Elements (IMEs) in the 400 strains of *Streptococcus suis* according to their phylogenetic clade, host and isolation site. Genomes have been grouped in clades according to their phylogenetic distance determined by alignment of the persisting genes as indicated by the phylogenetic tree at the left. The color code of clades and of other categories is shown on the bottom of the figure.

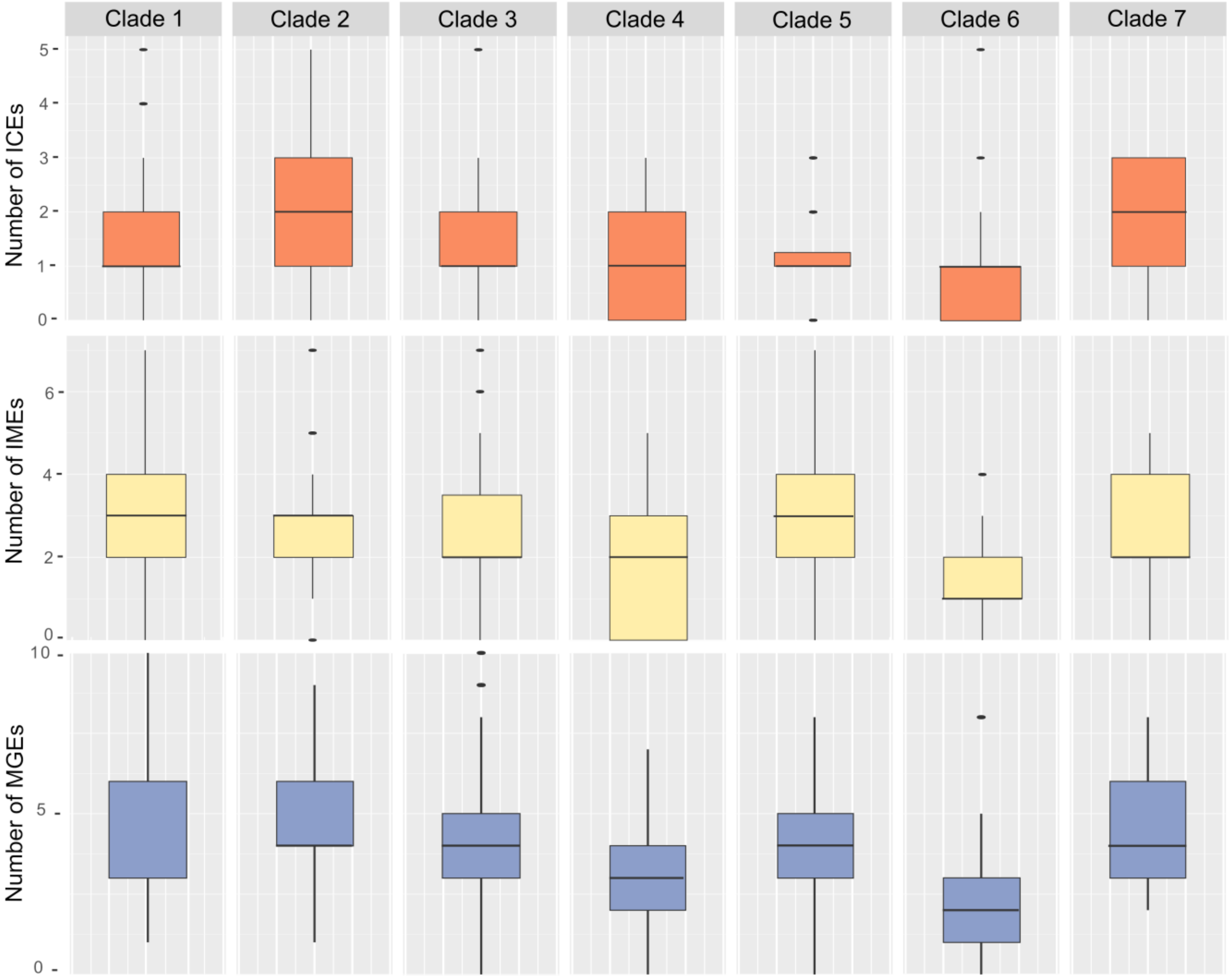

Figure S9: Number of Integrative and Conjugative Elements (ICEs), Integrative Mobilizable Elements (IMEs) and total Mobile Genetic Elements (MGEs) per clade. The box plots represent the data distribution with the interquartile range between the upper and lower quartiles, Q3 (75th percentile of the data) and Q1 (25th percentile of the data). The median values (middle 50% of the data dispersion) are indicated by a horizontal line inside the boxes. Outliers are indicated as black circles.

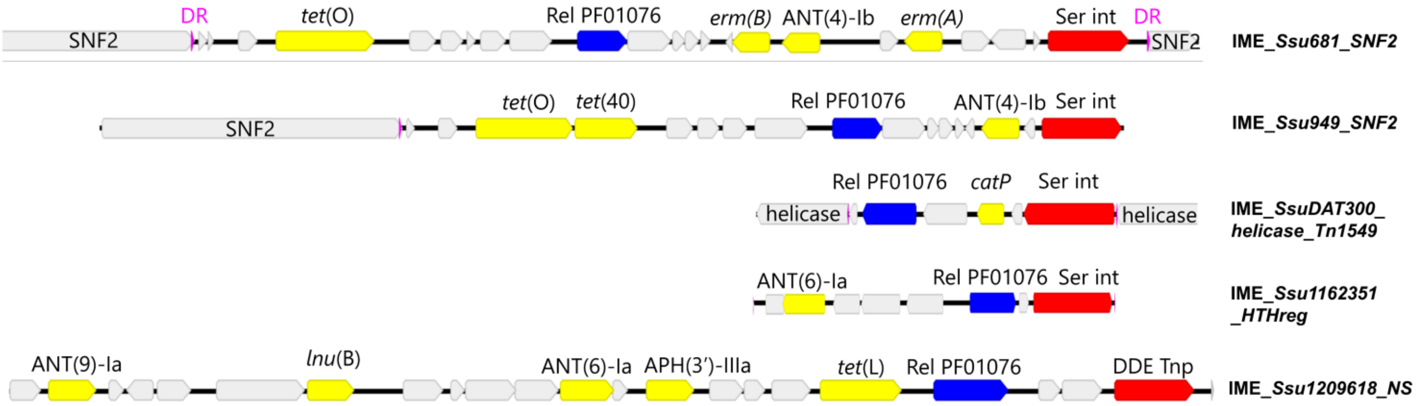

Figure S10: Examples of Integrative Mobilizable Elements (IMEs) with a MobV relaxase (that harbor a PF01076 domain) carrying antimicrobial resistance genes. The name of the IME indicated the name of the strain of *S. suis* and its integration site. Genes encoding a serine integrase or a DDE transposase are shown in red, those encoding the relaxase in blue. Antimicrobial resistance genes are shown in yellow. Direct repeats (DR) are indicated as pink vertical lines.

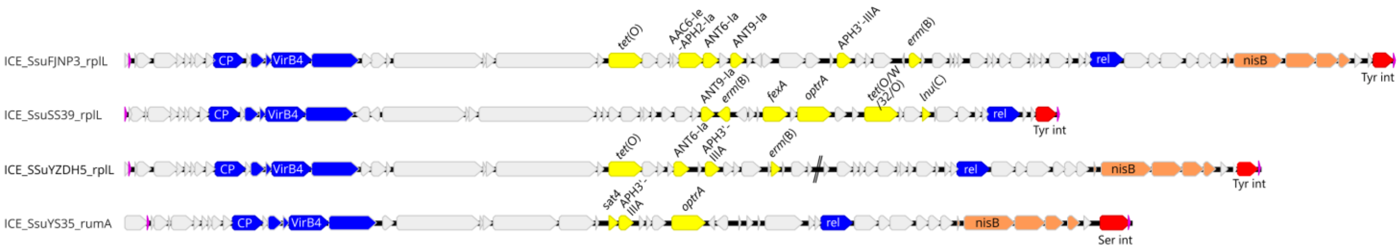

Figure S11: Examples of Integrative and Conjugative Elements of the Tn*5252* family carrying antimicrobial resistance genes. The name of the ICE indicated the name of the strain of *S. suis* and its integration site. Genes encoding the integrase are shown in red, the genes of the conjugation module in blue. Antimicrobial resistance genes are shown in yellow. Genes encoding an antimicrobial peptide are shown in orange. Direct repeats (DR) are indicated as pink vertical lines.

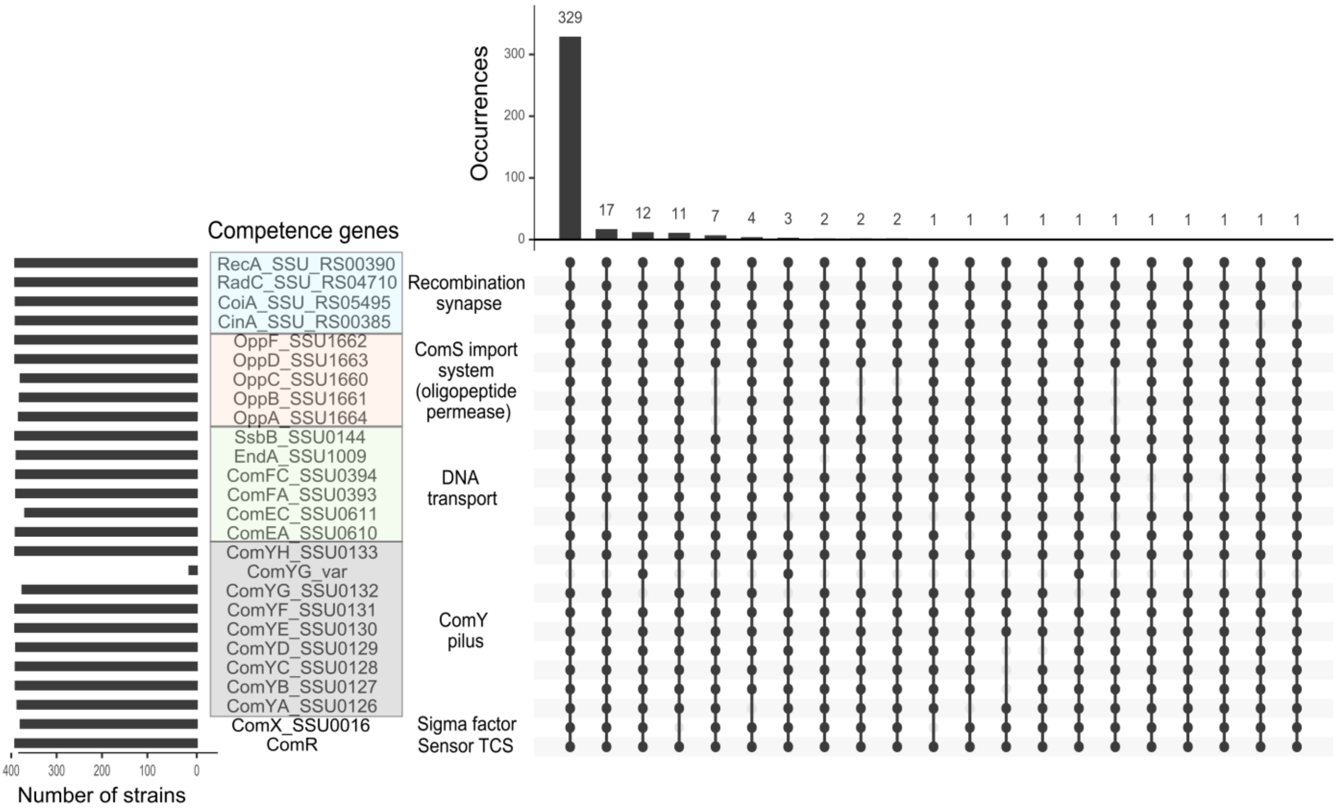

Figure S12: Occurrence and associations of genes required for competence in the 400 genomes of *S. suis*. The number of occurrences of each gene is indicated on the left with horizontal bars and the number of gene associations by vertical histograms (with indication of the number of representatives on the top of the histogram). Genes are grouped and coloured according to their function: genes of the recombination synapse coloured in blue, genes of the ComS import systems in orange, genes for DNA transport in green and genes for the ComY pilus assembly in light grey. Combinations of genes are indicated in the center of the figure by a circle in front of the genes, linked by a vertical line.

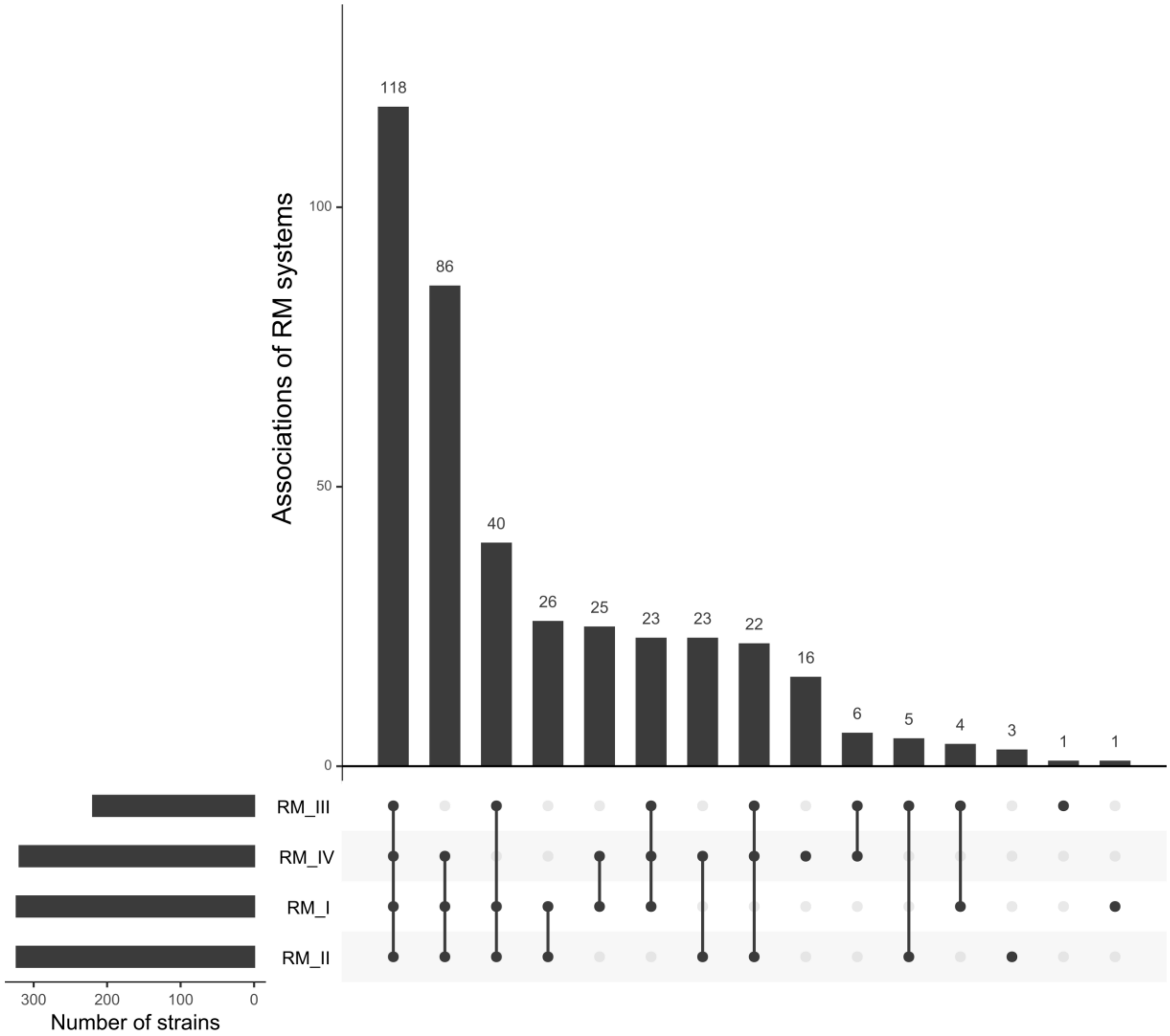

Figure S13: Occurrence and associations of Restriction-Modification (RM) systems in the 400 genomes of *S. suis*. The number of occurrences of each RM gene is indicated on the left with horizontal bars and the number of gene associations by vertical histograms (with indication of the number of representatives on the top of the histogram). Combinations of RM systems are indicated in the center of the figure by a circle in front of the systems in association, linked by a vertical line.

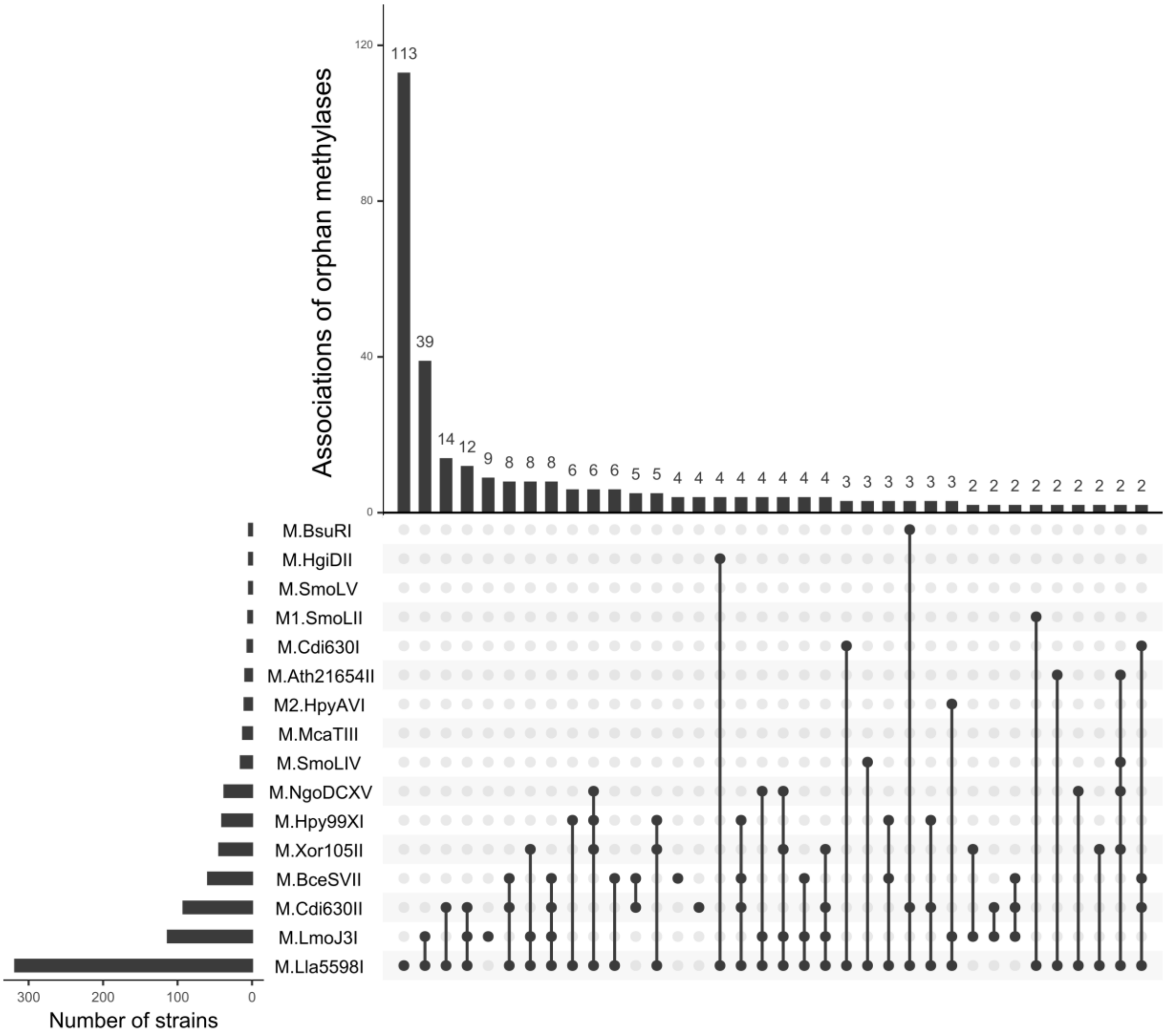

<u>Figure S14:</u> Occurrence and associations of orphan methylases in the 400 genomes of *S. suis*. The number of occurrences of each gene encoding an orphan methylase is indicated on the left with horizontal bars and the number of gene associations by vertical histograms (with indication of the number of representatives on the top of the histogram). Combinations of genes are indicated in the center of the figure by a circle in front of the genes in association, linked by a vertical line.

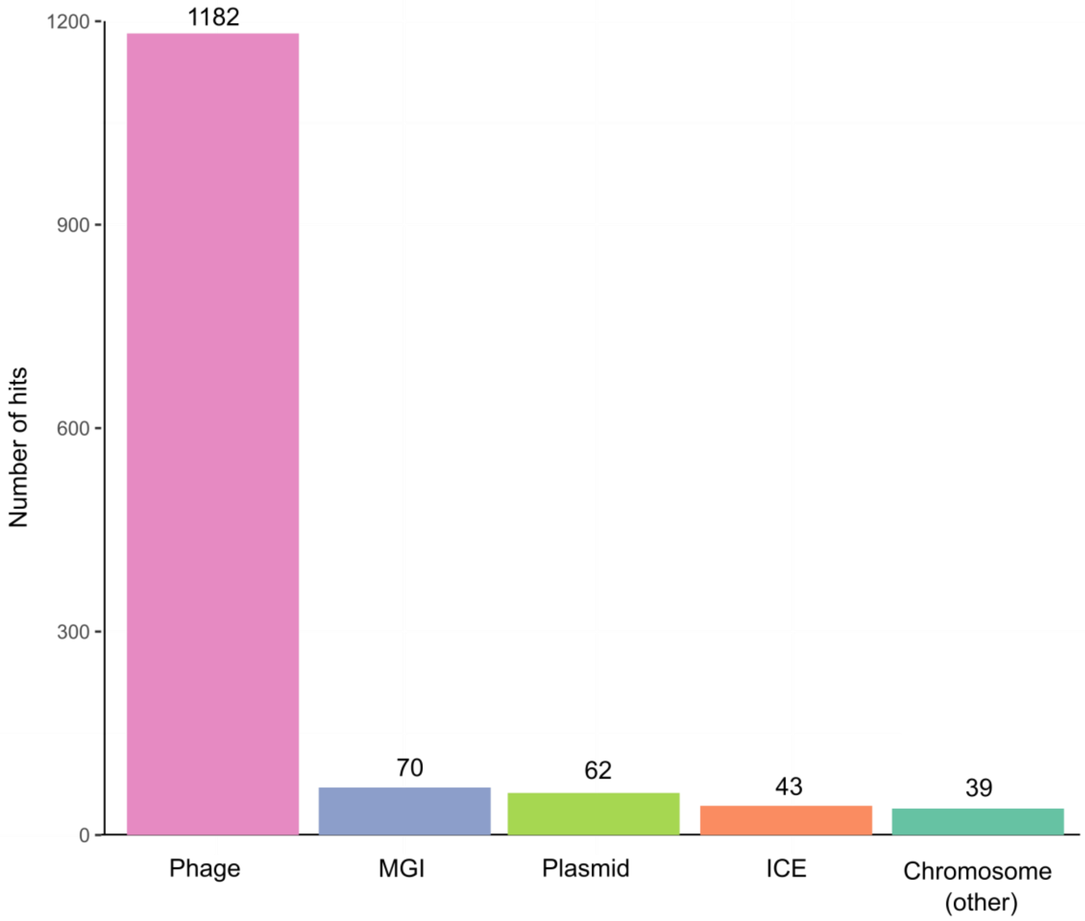

<u>Figure S15:</u> Number of spacers of CRISPR arrays targeting phages, Mobile Genetic Islands (MGIs) plasmids, Integrative and Conjugative Elements (ICEs), or self-targeting the host, indicated as « chromosome (other) ».

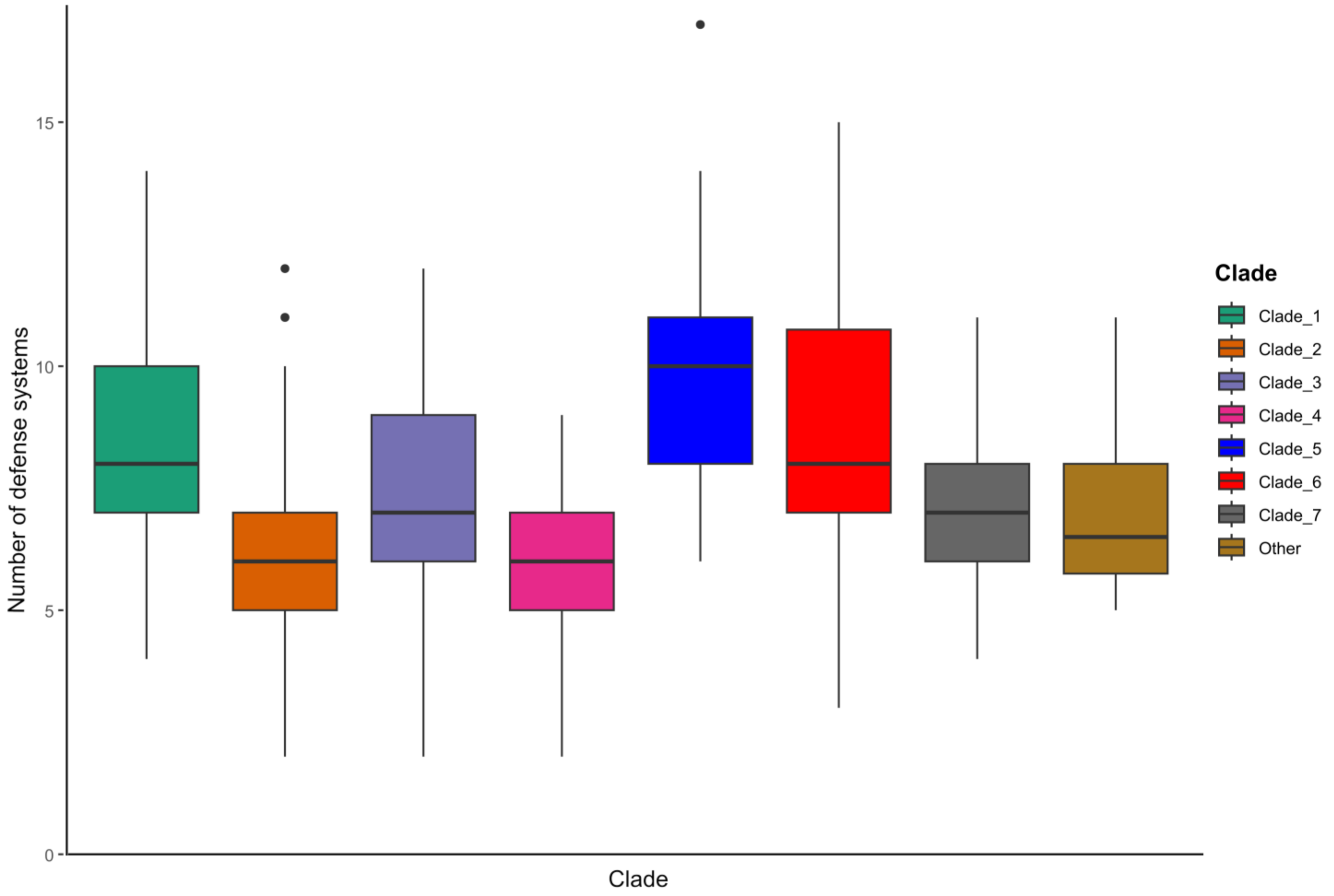

Figure S16: Number of defense systems according to the clade of the strains. The box plots represent the data distribution with the interquartile range between the upper and lower quartiles, Q3 (75th percentile of the data) and Q1 (25th percentile of the data). The median values (middle 50% of the data dispersion) are indicated by an horizontal line inside the boxes. Outliers are indicated as black circles. The boxes are colored differently according to the phylogenetic clade (see legend on the right).
