# Supplementary material for: Pan-immune system, mobilome and resistome in Streptococcus suis
Source: Microb Genom. 2025 Sep 29;11(9):001521. doi: 10.1099/mgen.0.001521 (PMC12479173; doi:10.1099/mgen.0.001521)
Supplement: Uncited Supplementary Material 3. [file mgen-11-01521-s003.pdf]

<u>Supplementary file S1</u>: Sequences of the *cps* locus used to determine the serotype of the *Streptococcus suis* strains.

>cps-1
AATCATGGAATAAAGCGGAGTACAGCTGAATATATTATGTTTGTTGACTCTGATGATGTT
GTTGATAGTAGATTAGTAGAAAAATTATATTTAATATTATAAAAAGTAGAAGTGATTTA
TCTGGTTGTTTGTACGCTACTTTTTCAGAAAATATAAATAATTTTGAAGTGAATAATCCA
AATATTGATTTTGAAGCAATTAATACCGTGCAGGACATGGGAGAAAAAAATTTTATGAAT
TTGTATATAAATAATATTTTTTCTACTCCTGTTTGTAAACTATATAAGAAAAGATACATA
ACAGATCTTTTTCAAGAGAATCAATGGTTAGGAGAAGATTTACTTTTTAATCTGCATTAT
TTAAAGAATATAGATAGAGTTAGTTATTTGACTGAACATCTTTATTTTTATAGGAGAGGT
ATACTAAGTACAGTAAATTCTTTTAAAGAAGGTGTGTTTTTGCAATTGGAAAATTTGCAA
AAACAAGTGATAGTATTGTTTAAGCAAATATATGGTGAGGATTTTGACGTATCAATTGT
>cps-10
TTACGAGGGGATTCTGGGGTGACCTTTGAGGATTTTTTAACTAATGCAACTTTTTATATA
AATGGTTCTCCTTTAAGTGCTACACTTTGGGAAACGGGGATTACTTTTACTTCTGTCAGC
AACGTTATCGATAAGGTTCCATCTGTTGTCCCG
>cps-11
TACAGTGCTTGCAGCCCTACTATTAATAACAATGGTATGGAATGCGATTGCAACAATTGA
CTTGCCCGACATGGAGAACTATTTTTGGTTATATCAAGGCCTGATAGGTGGTGTAGAAAC
TGGATATAGTTTTTTAATGGATTTATTCCGTAGCCATCAGTTTACTTTTTTACAGTTTCG
AGCAACAATATTAATTGTTTCGTATGTGTTGATTTGGTTAGGCCTCGTAAAAATAAAAGC
AAATAAAAACTTGGTGCTAGCCTTATATTTTGTTTTTCCCTATAATTTTGATGTCATTCA
ATTTCGTAATTTTTTTGCAGCTGCCATCTTTCTATACGGGCTTACTGATTTTATGCTTGA
TAACAAAAATGGCACAATTAAATTTGCCATAACTACATGGGGTTCTTCCCTCTTTCATTC
AATCAGTTATATTTATTTTATTCTGCTCTTAACGCATAAGAAGATAACGAATTTTAAATT
TATGAGACAATTATTTGTTGCCATTTTTGTCTCGTTGTTACTAGTATCCGTTCTCTTTAG
GGATCAACTTCTAGGTCTATTTTCAAGTATTTTTATTGGATTTAATCGCGAGAAAGCAAC
CTATTACTTGAATGAGGGAACAAGGAATGGATTTTTAGTTTTTTGGTTTTTCGAGATAGT
TTTCCTATATATATCATATTTGTTACATAACCAGAATAAAAAATTTAACTTTCTTTCTGA
GTTTGACTCAAAAGTTGTCGAAAAAATATTCTGGATTAATTTGATACTAACATTCCTATT
TCCTATAACAATGATTAATTTTAATTTTTCTAGGATTTTTAGAAATATATTAATCATCAA
CTATGGTTTAGGTGCCTATGTATTACTCATGCCTAAAATCAGGGCACGACAAGTCG
>cps-12
TGTGGCGATAGGACAACAGGTATTTCAGGATTGCTTATTTTAGCTCTTGTTTCATACAAC
AAAAATAGTGGGAATATTAAATATAATTGGAAAGATATATCTAAGATTAGCTTCATTGGT
CTATTGCTGATTATCTTTATCCGAGTAGTATATGCATATAGAACAGGAGAAGATATCCTA
TTTGCCTTATCAGGCGGAAACTTCTTGGT
>cps-13
CTGGTGCTGCAATTTCGCTTTATTCAACGGTATTGAATCAGGCATTATGCAATTCAATTC
CAATAATAATAGATAATATGAGTAATCCTGAAAATTTTAACAAATTGAAAGAATTGGGAT
ATGTTTGTCTTTATAAGGAGCATAAACTTTTATCAGAAGTAGTGGAGGAAAAAGCATGAT
AGGAACAATTATAAAAAAATTGAGAACAGATTTTTATACAAGTGTGTGTAAAAAAACAGC
TAAGCAATATGGAAAGGGATTGCATATAAACCATTGGAGTAAATTTACTTCTAAAACGTA
TATTGGCGATAATTGTCATTTTAATGGCATGAAGATTACCGGAAATGGAACTGTTACAAT
AGGTAACAATTTTCATTCGGGGGGGGGTATACTAATTTTAACATCTAATCATAATTATGA
GGGAAGAACAATTCCTTATGATAATACTATGATAGATGGTGATGTTGTTATTGAGGATAA
TGTTTGGCTTGGACAAAATGTCACAGTTTTACAGGGAGTTAAAATTGGAGAGGGAGCAAT
TATTCAAGCTGGGAGTGTTGTAGTTTCGGATATAGCAGCATGTTCGATTGCAGGAGGACA
TCCTGCAAAAGTATTCAAGAAAAGAAACGAAGAACATTATTATGAATTGAAACAAGAGGA
GAGTTTTTTTTGATTAACCAGTTAATATCTTGACCTCATTAGAATTAAAAGTATTAGAGG
AGCATTAATGTGAAGGTAAGATTAAGAATTAATAATGCAGCAATGATATATACATTGTTT
GCAATTGCTTTTATGGGTGGACATATTTTGAAAATACAGTCGCTTGTAGCGAATTTTGGT
ATTGCATTAGTTGGTCTTTGTGCATTTTCGTATAGTGTTTTGAAAAAAGGAATAAGGGGT
ATAAATGCATATCTTATCTTGATTGGTGCATTATATTCAGTATTTATGATACTTTCTCTT
TTTTATAATGGAAATGCTGATTATTTGGATCTGTTATGGATATGGGCATATATGGGCATT
GCTATTTTAACTTATGAATTTGAAATCTCTAAACGAACATATTGGAATGTTGCGTATTTT
ATTATAGCTTTAATATGTATCTATATGGTTAAAGGTGGAACTGCAGCTAGTCTGC
>cps-15
GCAAGAAAGCTTCCGGATGGAACTTTTCAATCGTTGGGACTAGTATTCGTTTATTCTATA
ATCATTTTTTTATTTCATAATCGTTTTGAGATTGAGTGGAGCATAATCACTTTATTAATT

TATCTCCCTCTTATACGTTCTGAAGAGAAGGGGTTTCTAAAAGGAATTATAACTATAAAA
TTGATTGTAGTTTCAATGATTGTAGCAGCAACTCTAATAGGAGTTGTATCTGATACAACG
CTCGTCAAATTTAATGGGGTTGCACACTCTCTTG
>cps-16
TGGAGGAGCATCTACAGCTCGGAATAAAGGGGTTTTAGAATCTCGAGGGGATTACATTGT
ATTCTGTGATGCAGATGATATGTATTTTCCTCATGCTATAGAAACTTTGTTGAGTGTGAT
GCAAAAGTATACTGCTGATATGGTAATTGGAGGTATCACCAATGTTGAATATAAAGAGGT
GCCTGAGATTCCAGCAAACAAA
>cps-17
ACTTGGGTTGGAATGGCGAAAAGTAGCACATACAATCCAGTTTATTTTGCGAATCAGTCG
GGTAGACAAAGATTAGTAAGGTTGATTAAAATTGGTATTATTTTGATTCTTCCTATTAAG
GTGTTGCTTGTTGTGTCCTCAATCCAGCTTATGGGGTTACCCTCGTTTAGCAATATATTT
TCAACATTAGCATCAGTTTATACGAGGCTGCATCACGGCGAAGAGACAGAGAACATTTAC
CGTCAGATAGATACTTTTTGTACAATGATATTTTACTTTTCGACATTTGCAGGTATATAT
TGGAGAAAAAAAAATGAAAACATTATTTTTCTTCTTGATTATAGTCAATATTTCACTTGAT
TTATTTTATAATTTGTACTTTATTGGGACACAGAGGTCTATCATAACAATTGTAGTTTTA
ATAATGACATTATTTGCCTGCAATTCAATTGAAAGTGATTTTCACATAAATAAAAGAAAA
CTTAAAAAGATTATATTAACAATTAGTGTGTTGTTAATTATATTTTTTAACATTTTATCT
GCAAGAAAAACACTTTGGGCTTCTTCCAATAGTTATATATATATGAATGAGCGTTTTGAT
TTCTACAATCCCCTACTTTTCTGGTGTGTCACAGATAAACTAAAATATGATGTGTGTAAT
TTGTTGTCATATTTTACACAAGGTTTCTATGGCTTGTCTTTATCCTTTCAAGTTCCATTT
GAATGGAGCTATATGTTAGGGAGTGTGAGAGGATTAAATAGTATTATATCTCAGTTCTTT
CCGTTTATCCCTAATATGATAGAGTTGACATATCCGTTAAGAGCAGGAGAGTCTTTTAAC
TTCGACGGTCTTGTTAACTGGTATTCGATATTTCCTTGGTTAGCTAGTGACCTGACTTTC
GGTGGT
>cps-18
CGGGGCAGTCTTACTCATGGAATGAAACATTGGCAACAATTTTTTCCCCTGTAATATTAA
ATGCATACTTTTCAGGACCCTACAATATGAATATTATTGAAGATGTAATTGAAAGAATAG
TGACTCTCGATATTTTTGTACCTAAAATTTTGATATCAGACTTATTTAATAATGCTCCGT
TTTTAAATAAGTTTTTGAGCGATAGGCTGTACACAACAGTTTCGTTATTTAATATTTCAT
TTTATAGTTCTACCGTTGCTACAGATCAGATTATCCCTCTCGCAGGACAACTTTATGTAT
ATTTTGGTTGGTTTTTTTGGATTGTAACTATTATCTTAACAAGTTTATCAATAAAATTGG
AAAAAGTTGCGTATAAGCAAAAAAACATTTTATTAATTTATGCTACGATGTTTCTGTCCG
TTTCGCTGTCAT
>cps-19
AGCAGGGTTGCGTATGGCGGGATTATGGGAGACTTTAGTTTTGCAACCCTGTACCTTGCA
TCAGGCTGTTTACTAATTCTGTTATTATATTTAAATGATAAAAGATTTAAATTTTGGTTG
TTTTTTATTTCTGAAAGTTTTTTGTTACTTTCAATGCTTGTTGTTTCTTCGCGAACTGGT
ATTGTTTCTTTGGTTATTACAGTTGTGCTTTACTTTATTTTTAATTTTAAAAAAATACCT
AAGCGCATGCTTGTTGTACTAGCTTTGGGTTGTACAGGAGTTCCGATTCTTATTGATAAA
ATTTTGTCCTCTAGAGGAGGACAAGCCTTATTAGAAACATCTGGTCGATCTGAAAATTAT
TTGCAATCGATTACTTTTTGGTTAGAGAAGCCTTTGTTTGGTTATGGATTAGGATTAGAA
AATTTATATCACTCTACCGGATTAGCAGTGCCTCATAATTTTTTTATACAATATTTATTA
CAAATGGGGCTTGTTGGATTGTTTCTAATTATTATACCATTCATCATTTTTATTACAAAT
GATGTCTTAAGGAATAATTATAGCAAGTGGTTGTTTTTATTAGTTGTAATTGGTTCGATG
TTTATTCCTGATATAGTGAGTTCTAGATTTTTATACGGAATCGTTTTGTTGTGTACGATT
GAGAATCGGATGTTCGACTATAATAATTTGGAGGTATTAAAAAGTGAAACGTAAGGCTGA
ATTGGTTGCTATTAAGGCAAGGAATTATTTTTTGGCAAAAAAATATAAGAGAGCAACTTT
TTTTTCTACAGTTTTGAGAGTGGTATTTTCTTGCGATTTACCAGCAGAAGTTCAATTTCA
TGAGTCAATTCAGTTGGTACATAATGGATTAGGTTGTGTTTTTCACCCAAAGACAGTCAT
CGCGGAGAATTGCAAAATATATCAGAATGTTACCTTAGGTGGAAATGGTAAGATTATAAA
CGGGGAAATCATAAGTGGCGCACCTAAATTAGAAAAAAACGTTGCGGTCTTTGCTGGTGC
TTGT
>cps-2
GATTTGTCGGGAGGGTTACTTGCTACTTTTGATGGAAATTATCAAGAATCTGAGCTGCAA
AAGTGTCAAATTGATTTGGAAGAGATAAAAGAGGTGCGAGACTTAGGAAATGAAAATTTT
CCCAATCATTATATGAGCGGTATCTTTAATAGCCCTTGTTGCAAACTTTATAAGAATATA
TATATAAACCAAGGTTTTGACACTGAACAGTGGTTAGGAGAGGACTTATTATTTAATCTA
AATTATTTAAAGAATATAAAAAAAGTCCGCTATGTTAACAGAAATCTTTATTTTGCCAGA
AGAAGTTTACAAAGTACTACAAATACGTTTAAATATGATGTTTTTATTCAATTAGAAAAT
TTAGAAGAAAAAACTTTTGATTTGTTTGTTAAAATATTTGGTGGACAATATGAATTTTCT

GTTTTTAAAGAGACGCTACAGTGGCATATTATTTA
>cps-21
GGTGGCAAGGAGAGCAAAGTATGCCAGAAGTTGTTAAATTGTGCTATCAAAGTCTACATA
ATTACTATGACCAGTCGAATATTTTTTTGATTACTCAAGAAAATGTTCACAACTATATTG
ATATTCCTGATTCAATATTAAAAAAAGTTAACAATAGAAACTTCACACTCACGATTTTTT
CTGATTACATTAGATTATCTCTATTGTCAAAATATGGGGGTATCTGGTTAGATTCTACAA
TTCTCTTGACTAATGCTCTAAACGAGGATGATAATGCTTATTTTTTTAGTATAAATTATG
GAAATCCAGCAATGGCTTACCATGT
>cps-23
TGCTCAACAAACGCAGCAAAAACATGGTTCGGTTTACTAGGTATTAGTAATTTTGAAATA
ATTCCCAATCCGTTAGAAGTAGAAAAGTATGGTCAAAATATGGATATTCGAAGGGAAGTG
CGTAGCCAATTAAATATAAAAGATGACACGATTATATTAGGACATGTTAGTAATATGACT
CCGTTAAAAGAAGTTCCATTTATTATAGACGTTTTAGATAGGTTGAGACAAGATGGCTTT
GAGTGTAAACTAGTCTTAATTGGTAAAGATGTTTTACCAGCAACTGTCAAAGATAAAATT
AATATTTATAATTTAGAACAAGAAGTGATTAATTTGGGAGTAAGAAGCGATATAGAAATG
GTGTTACATGCCATAGATGTATGTTTGATGCCTTCGAAATCAGAAGGGTTTGGTATGGTG
CCACTAGAATGCCAGGCTGCAGATGTACCAGTCA
>cps-24
ACCCGGAAAAACCAGGAGTTTATTACTCAAATTTACAAGTTGTGGATGAATTCTTAACAC
CTACTCACAAATTATTTAAAAAAGGTATTGTAAAAAATTCATTTGGACAATCATTAGCAC
AAGTTTTTTTATTTGCTTGTACTTCTGTGTTTAATAAGATTATGTTAGATGAGCTTATAA
AATATAATTTTTCTGATTTAGGTTTTGATTCGCTGTTATACTATCTTGGTATTTTAAATG
AAAATATTATATATGATGATACTCCCTACATTTTTTATAGGCAGCATGGACAAAATGTTT
CAGGTCAAAAGGAACAAGGGCTCAAGTATATAAAAAATAAGCTTATTCATTTTTTTAATC
AAAGTGAGAGAGCAGTTATGAAAAATAAAGCACAGTACATAGTAGAGAACCTGAGTCCGT
ATTTATCTGAGAAAAATTATTTATTAGCTCATCAAGTTTCAGCATACAGTGGTCTTTTAA
GTCGCTTGGCATTGATTGGT
>cps-25
GGAGGAGCTGCGGGCTCATACATTATTTTTGGTAATAAAGTCATTGAATATACAAAATAT
GCGATATTAATATATTATATAACCGCGATACCGATTATGCTTTATAATTTAGGAGTTGCT
AATTTTATAAATGGAGTATTGAGTCCTTTTACTGGTAGCTTAGTAACAAATCCTTTTGAA
CAAAATTCAGATTTGGTCTTATCTCTTGGTATATTAGTTATTTACTATTTTGACTATAGT
AAAGGTATGAAGAAAAGTCTATGGTTATTACCGCTAATGTTACTTATACTTGGTGGTAAA
AGAATTATGCTTCTATCCTTATTGATACTTTGTGGAATAAAAATATATAGCTCGATGATG
TCAATAAAGAATAAAGTACGATTGCAGTACTTTTTATCGTTTGTTCTCCTTGTAGCAATG
TTCATCTTTGTTTACTTGATCAAAAGTAGTATTTTTTCTAATTATGTTTATTCTCATGGT
ATAAATACAATGGGCAGGGTAAAAATGTGGGATTATGTAGCTCAGTATGTGGAATTTTCG
CCTTCATATTTGGGGTACGGCTACGCTTTTTCAAATCTATTGTTAGAACAAAATCGTGTA
CTAACATTTGGCAATAAGGTTTATGTACTTCATAGTGACATATTAAAGATTTATTATGAT
TTAGGTTTTTGGATTTTTACTTATTGGGGAATTTATAATTTATTTAGACTCCCGCATAAA
ATTGGAAAAAATTATAATCTTAAAATTGAAAATACAGTATGGCTACTCACAATCTATTTA
TTTTTACTATACTTTACGGATAATGCATTAACCTATTTTACAGTCCAGACTTTATATACT
TACACTGTTATCGATACTATCCGAAAGTATAATAGAGAATATAACTAATGTCTTGGTTTG
GAGTTTTATATGATAATAAAAGATATTATTTTTAATATAACTACTTACATTATAGCAAAA
ATATTTTTTAGAAAAAAAATATTGGTTAATTTCTGAAAGAGGAATAGATGCTAGAGATAAT
GGGTATTGGTTTTTTAAATATTTAAGAGAACAACATCCGCATATTGATGCGGTTTATGTT
ATTGATACTGATAGTCCTGATTATGATAAGGTTGCAATTTTGGGGAAAACAGTAAGACCA
AATAGCTTTTTACACGGTATTTTATTTTATGCTTCTGATAAAATAATAGGAACGCATCCA
GGTTGTGGCCA
>cps-27
CTACGCCAATCGAAGCCAGACACTGCTTGCATTATTGCTAGGGTTAATTCTATCATTCTT
TTTATATATAAATTATAAATTAAAGTGGCAAACATATACATATTTATTAGTTGCGTTAGT
TTCTTCTTTACTAAATCTTCTCTTCATTGAGGGGGCGGAATTTCTTTTAATTGGCTATGA
TTTGCTGATATGCTTTCCCATTGCGTTATATTTTGTTAAGGGAAACTATAATCGGTGGTT
TTGGAGGATATTTTCGATTGCTCTAGCAATTTTTGTAATAAATAAATATATCACGAGCAC
AGACATATTTAAGTATCAAATATTCCCTAATGCAAGTAGGAATTACATTTCAGTGTTTAT
GATTTTTGCAATGTTTTTATTTTGTATTTCCGAAGTAAAGTACAAGAATGCACTTAGAAA
CTTATTCGTATTTGCAGTTGTATCGTTTATGGCAAGTATTATGGCAGTTGGAAGGGGCGG
AATTATTGCGACAGGCTTCTTACTGG
>cps-28
GGACTTCGGTACCTTAGCGTTATTATTGCTCCTACCATTTGTAATCAATGAACAAGGAAA

GATAGTTCTCGAGAAACGCATAGTCTTTCCTTGGCTTCTATATGCGATATATATTGTAAT
AAGTCTTTATTTTCCGACTATTTTTGGAAAGGAAACTTATTTAACGAACGGTTCTATGTA
TGTGAGGGCTTTTAAGAATATTTTATATATATTTATTCTTATCTTAAGTACGTCCAGAGG
GATTATTAACAAAGATTATTTTGTACAGATTTATCTAAAGATAACTAAAATATGTACATA
TTATATCTTCATACAAGCTGCATTATATTATACTATTCGGTTCAACTTACCAGGGTACAT
CAATATTATGTTGGTTGCAGAAGGATATGGAGAACGGTTGGATGTGACGAGTTTCAGTCT
ATTTAGGCCGACTTCTTTATTTTATGAACCCGCTCATTTTTTTGAATATGTTGTCATTGG
TGTAATAATATATCTATTTAGATATAAAGAGACGCCTAAAAATGATATTTTTAATGCTAT
TTTCTTAGCATTAGGAATAGTCCTATCCACCTCTGGAATGGGTATTCTTACTATAGCGGG
TATTTTTGGTTTTTGGCTACTACAACAATTGAGTCGTACTAAGTTTATTCGTGTCAGAAA
AGAAATATTTTTTCTAACTGTAATAGGATTGATAGCAGTAGCATTCTTTCTACAGAGTGG
TTTTGGTCAAAGTGTTTTATCAAGACTATTTGATACAGGTACAAATTATTCTGCTTTAGA
TGGTCGTACTGAAATTTATGAAAGGATATTCAGTTTTGATACAGGTAGGGTATTACTTGG
CTCAGGGTACGGGAATGTGCTGGAG
>cps-29
GTGCGGGCGTTATTTTTGGTGCCTTTGTGTTGCCTTATCTTCTTGCTCTGTATAACAAGT
ATACCCATAAGAGATATATTATTGAGTTGTTGTTATGGGTGCCAGTCTTAATTATGATGA
ATAGTACAAGTGCGTATTTCATGGTAATTGCTGCCCTTATAGTATTCATCTTCATAGACT
ATCAAACAACTCAGAGACATAGCTTTGTTATTGCCTTCGTTGGCTTTCTTGTGCTGATGA
TCACTGTATTGAGTTTAGTTGGAGGAGCGAATATTTTATATCGTATCTTACCAAATGATA
TCAGCTATTTGATACTAGAAAAAACAACAGACTTAACAAATGGCTCCACTGTTTCTCGTT
TAGTTCCGATTGTCACCAACTGGAATATCTTTTTGAATTTTCCATTGCTTGGTGTAGGAA
ATGGGTTGCAAGGCT
>cps-3
TGGGAGAAGGCAGAAAGTACGAGATAGTTGGATAAGATGACAAAAAATAATAGAATCGCT
TTTTTAAATGATGTATTACTTAATTCATTATCTTTTTTCATTTACATATTAGGGCAACAA
TTGTTATTTATGCCATTGATGGGTAAATGGTTGACTGAAGGAATATATGCAAACTTTATT
ATATTTATTTCTGTTTTCGCAATACTATCAAATAGTTTGGGAAATGAGTTAGGTATAGTG
AGTCAGATTATTGAACAAAAAGTGAATTTTCAAAAAATTCTTCACTTTATTGCTTTTATT
TCTTTTTGTGTTACAGTGATAATTCTGTATTATCTTAATTTTAATTTTTTGGATACTGTT
CTTTTGTCTTTAAGTGTTTTTTTAGCAAATTATAGACTATTCTATTCCGGCTATTTTAGG
AAAAATTCTTTATTTAGAAATGTTTTAGTTATTAATATTTTATATTTAATTGGAATCTGT
TTTGGTTTGGCAGTGTATAGATTAACATACCTTATTTGGTCACCTTTGCTTTTAGCAGAA
CTTATTTCATTGATTTATATTTTTTGTTATCAAAATAAATTGGAAGAAGATAATATACCA
ATTTCTAAAGAAAATGTTAAAACATTTTTAAATTTTAGCTTAATATCTTTTCTGAATAAT
CTAATCACCTATCTTGATAAAATAATCATTTATCCAATATTAGGACCGACAGCAGTAAGC
ATATACTATTCCACTGCTTCTATGTCCAAAGTTGTAAATTTAGTTACTAACCCTTTGCAT
GGAGTTTTACTGAATTGGATAAAAAAATGATGGAAATAGAAATAATATTATAAAGAAATTT
ATTGTTGCAACCATGCCAATTATTGTAATAAGTAGTATCATTTCTATACCAATTACTTAT
TTTGCTATGAAACTACTGTATTCACAGTTTTTGCCCCAAGGAAATCAATTGATTGTACCA
GTATCTTTAGCTTTAGGAATTAGTATTGGAACTTCATTGTTGAAATCTGTTTTACTAAAG
TTTATTGATAGTAAAATTGTTCTTAGAATATTTATATTTTATTTTCTATCTTTTGTTATA
TTGGCATATGTTTCTAGTAATTTATTTGGATTAGTGGGCTTTAGTTATTCTGTATTTATC
TCAAAATGTATTTTGTTAATTGGGTTTATATCTCCACTTATAAAGCTTAAGGAGGCTAAG
GGTTGAAAAATATTACTGATACTTTAGTAAAAATTATTTATTTAAACATAATTCCTTCGG
CTCTTCTGGGGGT
>cps-30
CTTTAATTGCTTGCGCCCGTTGTGTGGAGTACCTAAATTCAGGTAATAGTCTGTTTTGGG
GAGAAAGTATATTTCAAATGTTCTATTCTTTTATTCCTCAGTCAATATGGGCAGACAAAC
CTACTCAGTATGGAATTATTACGTTGACAACTGCAATGGGTAGCCCGAAT
>cps-31
GGAGTGCTCTATGCCACCTTCAGTCTATGTTTAGTCTCTCTATATAGAACTTTGCAAAAT
TCTGAGACAGTTCTTTACTTGTCACGAAACAATGGAAAGAAGCAACCGCTACTACATAGT
TCAGATGCGATTATCTATCTGATAATGACAATCGTTTCTGCAATACGGTTAAATACAGGA
TCCGATTTTTATAATTATTATACCTATTTTGACCAAATATTGATACGATATAACTCATTT
GAAGAAGTCTTTTTTTCAATCACAGAGTGGGTATTTTGCTTTATCCTACATCATTAAGCAA
ATTACAGATTATCAGTACGCTATTTTTGCAGTTATTGCAGTATTTTCATATGCTTACCTT
TTTTATTTAATCAGATCAGAGGTAGAGGATACAACAAGTGCATTGTTAACGTATATGTTT
CTTGGCTATTATGCATATTCTAATAATATACTAAAGCAGTATATTGCAATGAATTTTGTA
ATGGCTACTTACCTCGCTTTGAGTAGAAGAAAATATTTTAAAACGATACTGTTTGCTGTA
GGGGCAATGC

>cps-4
ACTTGGAGTTGTCGGAGTAGTGCTTTCGAACATATTTGCTGAATTATTTGTAACAATTGT
TCGTCTCAAACCGTTAATAAATGAGTATGGATTGCCATTTAATTTTAGGGATGTTGTTGT
ATATATACTCTGTGGTATTAGCATTCTTGGAACTGGTTTTATTAATAATTTTATTATCCA
TTCAAATTTCTTATTCATCATATTTCAAACTTTTGTAGGAGCTTCTATATATTTTCTCTT
GACGTATATAGTAGGAGTTAATCCTGTCAAGAAAATTCTAAATAGAAAGTACGGATGATA
ATCAAATCTAGTTATTTATTTTGTATACATATTTTTATAGATTTTATAAGTTTATAGCTA
TGACTTAAGCTATTTGAAGTAAATAGAATAGTGGTTAATATTATAAAGTGTTGCAATTTT
CTATAACAAATGCTATACTTTGTACATCATTTGTTATAGAGGTGTTACAATGAGCAACAA
ATTTTTTTCATATGAAAAGTTGAAAAGCGTCATTGCTATTAAAAGAAAACAATTAGGTAA
TTCTCAACAAGAACTTGCAGATAAGTCAGGTATTAATCGTGCTATGATTAGCCGAATTGA
ACAAGGAAATTATATTCCATCTATTCCACAACTGGAAAGTTTAGCGCAAGTTTTGGAATT
TGATGTAGAATCGCTATTTATATCTAATAGTGAAGATAATAATGAAAATTCCTCAGTAGC
CAAGGTCGCAAAGATTAATATCGCCGTCGCAGGAACTGGCTATGTCGGCCTATCCATCGC
GGT
>cps-5
TGATGGCGGAGTTTGGGTCGCTCGTTATTTAGTGGGAAATCAGGAAATTGACCTGTTTGA
TAGAGTTAAAATTGGTTCTAATGTTCATATCGGCGTCAATGCTATCATACTGCCAGGAGT
AACCATAGGAAATAATTGTATTATTGCGGCTGGGGCGGTTGTTACG
>cps-6
TACGGTCTCCCTTGCCTGTACTGTTAATGATGTACCATGAAAGTGAACATAAAAAAACTC
AGGATGGTTTGGAGGTTATCAGGAGAGTTAAGGAGAAGTATCCGGAAATAGTAGTCAATA
TTTTTGGAAGAAGAATACCAGAAAAATTGCCCGATTCCTATAATGTTTTAATCAATCCTC
CAAGGGAGAAAATTTTTAAAATGTATCGAGAGTCGGATATTTATCTGTTTACAAGTGAGA
TAGAATCATGGGGTCTCCCAGTTGTGGAATCTATGGCTAATAAAGTAGCTGTAATAGGCA
GAAATCGTGGAGCACTAGCTGAGTT
>cps-7
GATGATTTATGGCACCCGAGTAAGCTAGAAAAACAGCTTGAATTTATGAAAAATAATGGA
TATTCATTTACTTATCACAATTTTGAAAAGATTGATGAATCTAGTCAGTCTTTACGTGTC
CTGGTGTCAGGACCAGCAATTGTGACT
>cps-8
ATGGGCGTTGGCGGGAGTTTCGATGTTTTATCAGGAACTATCAAGCGAGCTCCAATATGG
ATGCAGAAAGCGCATCTCGAATGGCTTTTCCGTGTTGCCAATGAACCAAGACGTCTTTTT
AAGAGATATTTTGTCGGGAATTTTGTTTTTATTAAGCGCATAGTCAAGGCAAAGAAAGAG
AAGAAATGAAATGAGTGAACAATTTGATATTAGAGAGCTCCAAGAAACGCTGTTAGATGC
TGTAAAAGAGTTCAAAAAAATTTGCGATGAGGAAAATATTACTTTCTTTTTAAGAGGAGG
CAGCGTGATGGGGGCCGTAA
>cps-9
GGGATGATTGCTCGACAGATGATACAATAAAAATAATAAAAGATTATATAAAAAAATATT
CTTTGGATTCATGGGTTGTCTCTCAAAATAAATCTAATCAGGGGCATTATCAAACATTTA
TAAATTTGACAAAGTTAGTTCAGGAAGGAATAGTCTTTTTTTCAGATCAAGATGATATTT
GGGACTGTCATAAAATTGAGACAATGCTTCCAATCTTTGACAGAGAAAATGTATCAATGG
TGTTTTGCAAATCCAGATTGATTGATGAAAACGGAAATATTATCAGTAGCCCAGATACTT
CGG
>CP011419.1_NSUI002_cpsK
ATGATTAACATTTCTATCATCGTCCCAATTTACAATGTTGAACAATATCTATCCAAGTGT
ATAAATAGCATTGTAAATCAGACCTACAAACATATAGAGATTCTTCTGGTGAATGACGGT
AGTACGGATAATTCGGAAGAAATTTGTTTAGCATATGCGAAGAAAGATAGTCGCATTCGT
TATTTTAAAAAAGAGAACGGCGGGCTATCAGATGCCCGTAATTATGGCATAAGTCGCGCC
AAGGGTGACTACTTAGCTTTTATAGACTCAGATGATTTTATTCATTCGGAGTTCATCCAA
CGTTTACACGAAGCAATTGAGAGAGAGAGAATGCCCTTGTGGCAGTTGCTGGTTATGATAGG
GTAGATGCTTCGGGGCATTTCTTAACAGCAGAGCCGCTTCCTACAAATCAGGCTGTTCTG
AGCGGCAGGAATGTTTGTAAAAAGCTGCTAGAGGCGGATGGTCATCGCTTTGTGGTGGCC
TGGAATAAACTCTATAAAAAAGAACTATTTGAAGATTTTCGATTTGAAAAGGGTAAGATT
CATGAAGATGAATACTTCACTTATCGCTTGCTCTATGAGTTAGAAAAAGTTGCAATAGTT
AAGGAGTGCTTGTACTATTATGTTGACCGAGAAAATAGTATCACAACTTCTAGCATGACT
GACCATCGCTTCCATTGCCTACTGGAATTTCAAAATGAACGAATGGACTTCTATGAAAGT
AGAGGAGATAAAGAGCTCTTACTAGAGTGTTATCGTTCATTTTTAGCCTTTGCTGTTTTG
TTTTTAGGCAAATATAATCATTGGTTGAGCAAACAGCAAAAGAAGCTTCTCCAAACGCTA
TTTAGAATTGTATATAAACAATTGAAGCAAAATAAGCGACTTGCTTTACTAATGAATGCT
TATTATTTGGTAGGGTGTCTTCATCTTAATTTTAGTGTCTTTCTGAAAACGGGGAAAGAT

AAAATTCAAGAAAGATTGAGAAGAAGTGAAAGTAGTACTCGGTAA
> CP024974.1_CZ130302_chzM
AATGAATAAGGAACTTGAACTAGATAATCCAAAAACATATAATGAGAAATTGCAATGGTT
AAAAATTAATGATAGAAATCCCTATTATACAGATCTTTCAGATAAATATAAAGTGAAAGA
TATTGTTGCTAATGCAATAGGAGGGAAATACATAGTTCCTACACTTGGGATTTGGGATAA
CGTACAGGATATTGATTTCGAAGCATTACCTAATCAATTTGTTTTAAAATGTACGCATGA
TTCAGGTAGTGTTATTATTTGTAGAAATAAAGAAGAGTTGGATATAAATAAAGTTAAAAA
AACTCTTGATAAAGCATTGAAGGGAAATTTTTATTACTATTCTAGAGAATGGTGTTATAA
AAATGTACAACCTAGGATTATTGCTGAAGAATATTTAGAAGATTTAGCTAATACAGATGA
TACG

<u>Supplementary file S2</u>: Sequences used to search for the CRISPR loci in *Streptococcus suis* genomes by Blast analysis.

>Cas9_Ssuis8830
MPNVFDEKQVKGLASFRKSKSQLFAKGWHNLSQKIMLEVIPELYATSDEQMTILTRLGKFEKSSVAEYPSSINVDEITDEIYNPVVA
KSIRQTIKIINASIKKWGEFDQIVIEMPRDRNEDEEKKRIADGQKANAKEKADSILRAAELYCAGKVLPDYVYNGHNQLATKIRLWY
QQGERCIYTGQPISIHDLIHNQNQYEIDHILPLSLTFDDSLSNKVLVLATANQEKAQRTPYNYLKSATSAWSYREFKDYVTKRKGIG
KKKCEYLTFEEDINGFEVRSKFIQRNLVDTRYASKVILNALQDYFKISGIQTKVSVVRGQFTSQLRHKWGIEKTRETYHHHAVDALI
IAASSQLRLWKKQESPLVVDYQEGRQVDLETGEILELTDEQYKELVYQPPYQGFVNTISSSAFDNEILFSYQVDSKVNRKISDATIY
ATRNAQLGKDKTEGIYVLGKIKDIYTQAGYEAFLKRYTKDKTSFLMYHKDLDTWEKVIEIILRDYREYDEKGKEIGNPFERYRRENG
YVKKYSRKGNGTAIKSLKYYDNKLGNHIDITPENSRNAVVLQSLKPWRTDVYFNKETGKYEFLGIKYSDLSFEKGTGEYGISQEKYD
SIKIAEGVAKKSIFKFTLYKQDLLFIKDIENNFGKLLRFTSKNDTSKHYVELKPYDKNKFGTEEPLLPVLGNVAKSGQCIKGLNKSN
ISIYKVRTDILGYRHFIKQEGEHPQLKFKK
>Cas1_Ssuis8830
MTWRIVHIHQSEKMRLKLDNLVIKKQGDEFTIPLSDISIIVAEGGDTVVTLRLLSALSKYNIALIVCDNHHLPTGIYHSQNGHFRAY
KKLQAQLSWAPLQKDKLWQIIIYFKITNQQDVLAMQEKSLSTIQLLSDYREHIELGDRTNREGHAAKVYFNELFGKQFVRLTQQETD
AVNAGLNYGYTIFRAQMARIIAGYGLNALIGIFHKNEYNQFNLADDLMEPFRQIVDVWVYMNLREADFLTYQHRLELTNLLNAKIRY
GKENCSVTVAMDKFVKGFIRCIEDKDTNQFFCPVVSSLEMEKL
>Cas2_Ssuis8830
MRYDAMRLLCFFDLPMETTQEKRQYRLFRKELIANGFEMLQFSVYYRTCPNRSFATKFYKKLQQSHLPAGNVRLLAVTEKQFSEMVL
IVGGKTRQEEAISDRKLVII
>Cas7_Ssuis8830
MKWLITHHVLNNIPIHVGQFTQIVGQNYELKYYIWQILIWYFGGKKYSEEDLILFNQEEPVISQDSEAIKRNAFKIISIAEVGDILE
QISYKKGTIGFSYLSLKMQNIEIIEELEILNYHLHKIAQKVNSSISLINDEIEYEVGTTDLLPEQILTKQLTPHFKKSRDEIAIEFI
SNEKKLCFLLQMLNAIMQEQTKPILLVLKNLDDYLTYDSFVRIAQYLEELSNKYPYFNTILFPSQEGYLYLTEATLETVNIVSDRIE
HYPAFTFLYTRYQQSYPSTSPLGEKEFLNSLRKISSYLFSSDINRVVSLADIDLVTLKIVNSLYQYDIKMVYMYKGISKLEENYLSS
>Cas3_SsuisNCTC10446
MNLAHIEKRERESRFQYLEDHLFNVAKEAKASAETIGQGDILFLLGLYHDLGKSDRLFQKKMKEEPSLHVDHSYAGARYLFQEIEQV
FRRSGKTSNDAQLFREIIAYIISAHHGIYDVPLPEDMEGAGRYRYSKLFYRIGQPRADYHYEEDIRAYAKLLEEQLPSFGYQNVDDL
VTKSFGNFQTAWAKLCINDDSESAFYSSCFIRLYLSYLKNADILDTINAYDLVIEPKTTEENSELVQKYFQSVEEVYAGFGNPTTEL
NRIRTALGERVKQRGATDSAGIYRLNLPTGAGKTNLSLRYAVHQLTQKGKKSFFYMTPFLSVLEQNAAAIQEIIGKEGVLEHHSNLV
REQQDTIPQNEEYGEETVNSLMTDYLIDTWDSPVVLTTMVQFFQTLFKTKSANIRRFSSLANSILILDEVQSLPIEVTTLFNLTMNF
LSRVMGATVVLCTATQPAYDSVSISHRLLYGGEIAEQADIVTLTAEEQEVFKRAELHKFDESNAVSRLSDLASFILEEETSTLVILN
TKLAVEKLYSLLEFQTDRPLYHLSTNMCAQHRLDVIQEIKNQLKDDVPLICISTQLIEAGVDVDFERVVRSYAGLDSIVQAAGRCNR
EGKRDLGRVTLINLSKEEESLTYLKEIKHKKEATETILIKESSPIDVGALNRLFFERYYADNAKQFDYPLSTNESVYDYLSLSSFKG
SPNCKVRQSFKMAGQKMDLIKDDSIGVLVPYGDGADIIANLEERLVDTPYPRGQDLVEIKQTLKSLQPFTVNLRQYDSRLQAVRYYL
EGQVLILQEEYYDDEKLGLKKEANLPIL
>Cas5c_SsuisNCTC10446
MYRSRNFYLRVKGDLALFTNPATKGGGERSSYAVPTRQALKGVVDAVYYKPTITNVVTEVKIVNQIQTELHGVRALLANYGADLSYV
SYLSNVEYLVKFHFIWNENRPDLVHDRLPNKHEAIMERSIKKGGRRDVFLGTRECLGLVEAISQEEYETAKLEYADKTIDFGIMFHS
FAYPIESNQPLRSYFTKTVMENSVIRFKEQEDCEVVNTLSNYVFKTSGDIKSVEEEFEIYSRDQENEEGGL
>Csd1_SsuisNCTC10446
MDFFTALLRAYEAAEDTGWVDNAQRSTNPLLPIYHTSRRSNGKDTIAVLLDNEGNFMKADFMADGESIIFPVTSDSVARSGKNPAPH
PLVDKLTYYLSEINQEQYDTYHRQLDEWISQCQEPEVKKFLSHIQHFLLQDDFIEKIVQSLYGRVTSREGLKVTFLNSDDSKKTVDL
STAFLEFKLDQFTGYQTVSVTNYLDLHKAYISYVESNQQADIICNISGKQEVLAAKHRGLIGNAKLISVSNNIETYKGRFKEREDVF
TVGNQTSEKIHLMAKFLLENEHTHARLGSGQHLINWFSDDLINETQLDITTPKVEEIEDAGIFDFGETSSDDTPKFQITEKNKKIRL
SFIHGKKEFGDGATYYVAIVNKTNDGRVALKYFRQLAASQLLDNLNKWQDKYSWQFKKKNGEYAECPPSYIDIILAAYGVDRGRFLE
LDNDKFKSDQFQKLATSMIDGKDVPDTILGKLKDNIKQRQRYGNTWNKVLFVSLALLHKTNKEEFTPMLDHKNKNRSYLFGRLLAIF
ELLELQRYQLDGSKNDRIANAERYWTAYTSQPAKLMMNLTNKIKPYEETVKLNSPGIFNKLEKEREEIMGLLGPLMQERGINAPLDY
RFIFGYYAEKQYFYTKQEKTESEK
>Csd2_SsuisNCTC10446
MLEQKIDFMVTIEVREANANGDPLSGNMPRTNAKNQGIISDVAIKRKIRNRMQDMGHKIFVQANDRIEDGLNSLEKRFKAQFSGKES
DEEINEKANQIWLDVRSFGQVFTYLKDRSFAIRGPVSVSMAHSLEPIIISSLQITRSTNGVEPKKAGGRSSDTMGTKHFVDYGVYVI
KGSINAYFAEKTGFTAEDAEVLKETLITLFENDASSARPEGSMRVREVFWFTHSNKLGNVSSARVFDLLEFDEEKQDKSSYEDYAIG
LKKAELDEFQVKGLTVEILEGL
>Cas4_SsuisNCTC10446
MTYAEDDYLMLSGIQHFQFCKRQWALIHIYQEWAENEATTHGQFLHQKADNPYIKEKRKDFLISRAMQVSSKELGLYGILDVVEFHK
DEAGISLSGKRGKWIPTIVEYKRGKPKKDERDIVQLVAQTMCLEETLACKIDKGYLYYHSVNKKVEISISAELRRLVIRLASQMHEL
YQKRDLPKAEYFKNCQLCSLVDICMPRLSKKSRSVANYIQQSMASEEGL
>Cas1_SsuisNCTC10446
MKKLLNTIYLTQEDFYLTRERDNIVIKQNGIAVHRFPYRIIDGIVCFSYLGASPSLIELCAEHQINLSFHTPQGRFCGRFVGPTNGN
VLLRRQQYRLADDALSLEFAKRFILSKISNSRKYLLRFRRDHKDRVDGNLFEEVNTELTWAVEMVQTALDKETLLGIEGQAANHYFR
LFNEMVLADKEMFQFNGRSRRPPLDCVNALLSFGYSLLTYECQSALEAVGLDSYVGFFHTDRPGRASLALDLVEEFRSFIVDRFVFS
LINRGQLTKKHFDIKENGSVLLTEKGRAVFIEAWQKRKHTEVEHPFTQEKVKLMLLPYVQAQLLAKAIRGELDSYPPFMI
>Cas2_SsuisNCTC10446
MVLVTYDVNTETVAGRKRLRKVAKLCVDFGQRVQHSVFECSVTPAEFVEVKNRLLEIIDKEQDSIRFYMLGKNWQNRVETIGRDTSY
DPDQGVLLL

>Cas9_Ssuis6407
MVKKKYAIGIDIGTNSVGWSVVTDDYKVPSKKMKVFGNTEKRYIKKNLLGTLLFDEGNTAENRRLKRTARRRYTRRRNRILYLQEIF
AEEINKIDDSFFQRLDDSFLIVEDKQGSKHPIFGTLQEEKEYHKQFPTIYHLRKQLADSSQKADIRLIYLALAHIIKYRGHFLFEGD
LKSENKDVQHLFNDFVEMFDKTVEGSYLSENLPNVADVLVEKVSKSRHLENILHYFPNEKKNGLFGNFLALALGLQPNFKTNFELAE
DAKIQFSKETYEEDLEELLGKIGDDYADLFIATKSLYDGILLAGILSTTNSTTKAPLSSSMVNRYEEHQKDLALLKNFIHQNLSDSY
KEVFNDKLKDGYAGYIEGKTTQENFYRFIKKAIEKIDGSNYFIEKIEREDFLRKQRTFDNGSIPHQIHLREMQAIIRRQAKFYPFLE
ENQDKIEKILTFRIPYYVGPLARGKSEFAWLNRKSDEKIRPWNFDEMVDKETSAENFITRMTNYDQYLPDQKVLPKHSLLYEKFAVY
NELTKVRYVTEQGKSFFFDANMKQEIFDRTFKVYRKVTKEKLMDFLSKEFDEFRIVDLLGLDKDNKSFNASLGTYHDLKKIVSEDFL
DNSENEDILENVVLTLTLFEDREMIRKRLEKYKDVLTEEQRKKLERRHYTGWGRLSAKLINGIRDKVTRKTILDYLIDDGTSNRNFM
QLINDDTLSFVDEIRLAQGSGKAEDYRAEVQNLAGSPAIKKGILQSLKIVDELIEVMGYDPEHIVVEMARENQFTNQGRRNSQQRYK
KIENAIKNLDSNLNSKILKEYPTNNQALQNDRLFLYYLQNGKDMYTDEELDIDQLSQYDIDHIIPQAFIKDDSLDNKVLTKSAKNRG
KSDDVPSLEIVYKKKNFWKQLLDSQLISQRKFDNLTKAERGGLTNEDKARFIQRQLVETRQITKHVARILDTRFNTKLDEAGNRIRD
PKVNIMTLKSSLVSQFRKDYQLYKVREINNYHHAHDAYLNAVVATALLKKYPQLAPEFVYGDYPKYNSYKSRKSATEKVLFYSNIMN
FFKRVLVYSKTGEVRIRPVIEVNKETGEIVWDKKSDFRTVRKVLSYPQVNVVKKTEVQNHGLDRGKPKGFYNANLSPRPKEGSVENL
VPAKQSFDTKRYGGYAGISNSYAVLVNGIIEKGKKKSKAEVTEFQGISILARKDFDKNPKQYLLNLGYKDIKSIIKLPKYSLFELEN
GSRRMLASILSTNNKRGEIHKGNEMYLPDKFVTLLYHAMRVNRTLEPGHKKYVEAHRHVFDELLTYILEYNEKIVGAKANGKRIVEA
YSARKDTDNLEELCNSFINLLNLTALGSAVDFEFLGTKIPRYRDYTPSSLLKATLIHQSVTGLYETRIDLSKLGGD
>Cas1_Ssuis6407
MGWRTVIVNTHSKLSYKNNHLIFKDATRTEMIHLSEVDILLLETTDIVLSTMLIKRLVDENILVIFCDDKRLPTAHLMPYYARHDSS
LQLSKQIDWEEAVKAEVWTHIISQKILNQSIYLSACGFIEKSQSVMNLYHSLELFDPSNREGHSARIYFNTLFGNDFNRELDNDINA
SLDYGYTLLLSMFAREVVLSGCMTQLGLKHANQFNQFNLASDIMEPFRPIIDQIVYANRNHSFVKIKRELFTIFSDTFQYNNKEMYL
TNIVSDYTKKVIKALNNKGKGVPEFRI
>Cas2_Ssuis6407
MSYRYMRMLLMFDMPTETVDERKAYRKFRKFLINEGFIMHQFSVYSKLLLNNSASNAMLERLKANNPKKGNITLLTVTEKQFARMIY
LHGQRDDCIGNTDSRIVFLGEEI
>Csn2_Ssuis6407
MKLNFPLLDEALTVEKATIFVVEDTTVFSRLVRNLYQYQDGLELKIFDEQFRSIKDSELMVVTDILGYDINAAPILKLIHADLENQL
NEKPEVKSIIEKLANSITELISYECLENELDLEYDEITVLELIKALGVKIETISDTIFDKIFEILQVYQFLNKKRFLVFINVLSYLT
VDEIQKTREYIELSNMDVLFLEPRKRKDFPQYVLDKDYFLLSENMVK
